# Use of Dairy and Plant-Derived Lactobacilli as Starters for Cherry Juice Fermentation

**DOI:** 10.3390/nu11020213

**Published:** 2019-01-22

**Authors:** Annalisa Ricci, Martina Cirlini, Antonietta Maoloni, Daniele Del Rio, Luca Calani, Valentina Bernini, Gianni Galaverna, Erasmo Neviani, Camilla Lazzi

**Affiliations:** 1 Department of Food and Drug, University of Parma, Parco Area delle Scienze 49/A, 43124 Parma, Italy; martina.cirlini@unipr.it (M.C.); antonietta.maoloni@studenti.unipr.it (A.M.); luca.calani@unipr.it (L.C.); valentina.bernini@unipr.it (V.B.); gianni.galaverna@unipr.it (G.G.); erasmo.neviani@unipr.it (E.N.); 2 Department of Veterinary Science, University of Parma, Strada del Taglio 10, 43126 Parma, Italy; daniele.delrio@unipr.it

**Keywords:** lactic acid bacteria (LAB), cherry juice, fermentation, dairy and plant isolates, volatile and phenolic compounds

## Abstract

Background: Lactic acid bacteria (LAB) exhibit a great biodiversity that can be exploited for different purposes, such as to enhance flavours or metabolize phenolic compounds. In the present study, the use of dairy and plant-derived LAB strains to perform cherry juice fermentation is reported. Methods: The growth ability of *Lactobacillus plantarum*, *Lactobacillus casei*, *Lactobacillus paracasei* and *Lactobacillus rhamnosus* was studied in cherry juice. Profiling of sugars, organic acids and volatile compounds was performed by GC-MS (Gas Chromatography-Mass Spectrometry), while the phenolic fraction was characterized using UHPLC (Ultra High Performance Liquid Chromatography) equipped with a linear ion trap-mass spectrometer. Results: Sucrose significantly decreased in all fermented samples as well as malic acid, converted to lactic acid by malolactic fermentation. The total amount of volatile compounds increased. Specifically, propyl acetate, an ester with fruit notes, reached the highest concentration in *L. rhamnosus* and *L. paracasei* (dairy strains) fermented juices. Phenolics were extensively metabolized: caffeic acid was converted into dihydrocaffeic acid, *p*-coumaric acid into 4-ethylphenol and phenyllactic acid was produced. Conclusion: Lactic acid fermentation confer fruit notes to the juice and enhance phenyllactic acids, especially employing dairy strains (*L. rhamnosus* and *L. paracasei*). The level of dihydrocaffeic acid, a compound with putative biological activity was also increased (in particular with *L. plantarum*).

## 1. Introduction

Sweet cherry, *Prunus avium L.*, is a native tree of Europe and western Asia and grows wild around the world [1]. Its fruit is consumed fresh, but also after processing, as canned, dried, frozen, and in syrups and juices. The main producers of sweet cherry are Turkey, United States, Iran, Spain, Italy and Chile. In Italy the annual production is about 100,000 tonnes, and the Apulia region is the most relevant area of production [2]. Sweet cherry is characterized by a high content of micronutrients and bioactive compounds, even if these attributes strongly depend on cultivar, ripening, growth condition, pre and post-harvest treatments [1]. Sugars and organic acids in fruit have been reported in the 125–265 g/kg and 3.67–8.66 g/kg ranges on fresh weight basis, respectively [3]. The balance between sweetness (sugars, mainly glucose and fructose) and sourness (acids, mainly malic acid) is paramount for the acceptance of the product by consumers [4], as well as the fruit aroma, despite the fact that aromatic compounds represent only 0.001 to 0.01% of the total fruit weight [5]. The aroma of cherry is related to a wide number of organic compounds, including aldehydes, alcohols, esters, acids and terpenes [6]. Aldehydes and alcohols represent more than the 80% of total volatile compounds, followed by acids, esters and terpenes. In particular, the most represented compounds are hexanal, (E)-2-hexenal (green note and fresh green odours), 1-hexanol (floral and grape notes), (E)-2-hexen-1-ol (vegetable note), benzyl alcohol (floral note), and benzaldehyde as the most important contributor of the typical cherry note. Moreover, the aroma of sweet cherry fruits is also influenced by non-volatile glycosidically bound precursors that, in sweet cherry, are more concentrated than the free forms. Glycosylated forms of alcohols, terpenes, norisoprenoids and organic acids are well known, and it is now accepted that the release of these compounds could strongly modulate fruit flavor [7,8,9].

Cherries are also rich in polyphenols, compounds derived from secondary plant metabolism and characterized by one or more hydroxylated aromatic rings: anthocyanins, phenolic acids, and flavonoids are the main phenolics observed in cherries.

Lactic acid bacteria (LAB) are the most widespread microorganisms involved in food fermentation, and their ability to convert phenolic compounds has been reported in literature [10,11,12,13,14]. The field of fruit juice fermentation represents a new interesting ever-increasing line of research for product innovation [15,16], although the number of fermented commercial products is still limited. The starter strains generally used for fruit fermentation belong to *Lactobacillus plantarum* species, recognized to be the most suitably adapted for these types of substrate, and autochthonous strains have often been used for the same reason. Lactic acid bacteria exhibit a great biodiversity, also derived from their ability to adapt to different environments, and this biodiversity can be exploited for different purposes, such as to enhance flavors [17,18]. Based on these assumptions, in the present work, the contribution of different LAB species, isolated from dairy and plant products, were evaluated in the framework of cherry juice fermentation. Although *L. plantarum* was already used to ferment cherry juice [19], the novelty of this study was to evaluate the contribution of different LAB species isolated from dairy and plant products. The ability to adapt to this specific matrix, the metabolism of sugars and organic acids, and the effect on the fruit volatile and phenolic profiles were investigated considering a large set of dairy strains as starters.

## 2. Materials and Methods

### 2.1. Chemicals

Analytical standards of 3-*O*-caffeoylquinic acid, 5-*O*-caffeoylquinic acid, 4-*O*-caffeoylquinic acid, protocatechuic acid, quercetin-3-rutinoside hydrate, quercetin dihydrate, kaempferol, phenyllactic acid, caffeic acid, naringenin, (+)-catechin, (−)-epicatechin, *p*-coumaric acid and toluene were from Sigma-Aldrich (St. Louis, MO, USA). Dihydrocaffeic acid, *p*-hydroxyphenyllactic acid were from Santa Cruz Biotechnology (Santa Cruz, CA, USA), while luteolin was from Extrasynthese (Genay, France). HPLC-grade acetonitrile was from Sigma-Aldrich (St. Louis, MO, USA), while HPLC-grade water and LC-MS grade formic acid were purchased from VWR International (Milan, Italy).

### 2.2. Bacterial Strains

Fourteen strains belonging to different species of lactic acid bacteria (*L. plantarum, Lactobacillus rhamnosus, Lactobacillus casei* and *Lactobacillus paracasei*) were singly used for the fermentation of a commercial cherry juice (Table 1). All bacterial strains were stored at −80 °C in de Man Rogosa and Sharpe (MRS) medium (Oxoid, Milan, Italy) supplemented with 25% glycerol (*v/v*). The cultures were propagated three times with about 3% (*v/v*) of inoculum in MRS and incubated in anaerobiosis (AnaeroGen, Oxoid, Basingstoke, UK) overnight at 30 °C for *L. plantarum* and 37 °C for *L. rhamnosus, L. casei* and *L. paracasei*.

### 2.3. Fermentation Process and Storage

A commercial pasteurized cherry juice (Bionaturae) was used for fermentation. The absence of microbial contamination in the juice was evaluated on Plate Count Agar at 30 °C and 37 °C. LAB strains were cultivated in MRS broth for 15 h at 30 °C for *L. plantarum* and at 37 °C for *L. rhamnosus, L. casei* and *L. paracasei*, to reach the late exponential growth phase, centrifuged at 10,000 g for 10 min at 4 °C, washed twice with Ringer’s solution (Oxoid, Milan, Italy) and re-suspended in sterile distilled water. These cultures were individually inoculated into cherry juice to reach the final concentration of ca. 7 Log CFU/mL. The juices were incubated at 30 °C for *L. plantarum* and at 37 °C for *L. rhamnosus, L. casei* and *L. paracasei* for 48 h and then stored for 12 days at 4 °C. Unfermented cherry juices (not added of starter cultures) were incubated at 30 °C and at 37 °C for 48 h, then stored for 12 days at 4 °C and used as controls. All the fermentations were carried out in triplicate.

### 2.4. Evolution of Bacterial Growth and Acidification of Cherry Juice

Cherry juice, inoculated with starters, was analyzed before and after fermentation (48 h) and after the storage period (12 days). Cultivable cells were determined using the standard plate count agar method as follows: decimal dilutions of samples were carried out in Ringer solution (Oxoid, Milan, Italy) and plated on MRS agar, then incubated at 30 °C (*L. plantarum*) and 37 °C (*L. rhamnosus, L. casei* and *L. paracasei*) for 48 h under anaerobic condition. The pH of samples was measured using a pH metre (Mettler Toledo, Greifensee, Switzerland). Plate count and pH measurement was carried out in triplicate.

### 2.5. Sugars and Organic Acid Analysis

Sugars and organic acids were analyzed after fermentation and storage, both for fermented juices and for controls. The method reported by Cirlini et al. [20], with slight modifications, was applied. Briefly, 10 µL of samples was added to 1 mL of a solution containing two internal standards (turanose and glutaric acid 500 µg/mL each), dried under vacuum and dissolved with 500 µL of dimethylformamide. Subsequently, silylation was carried out adding 400 µL of hexamethyldisilazane and 200 µL of trimethylchlorosilane to the samples and heating for 30 min at 70 °C. Samples were analyzed by a Thermo Scientific Trace 1300 gas chromatograph coupled to a Thermo Scientific ISQ single quadrupole mass spectrometer equipped with an electronic impact (EI) source on a BP5MS capillary column (30 m × 0.25 mm, with 0.25 µm film thickness, SGE Analytical Science, Milan, Italy). Chromatographic conditions were the following: initial oven temperature, 60 °C, then increase of 20 °C/min up to 280 °C; carrier gas, (flow rate, 1 mL/min). Temperature of the transfer line was maintained at 280 °C, while the ion source was set at 230 °C. The acquisition mode was full scan (m/z: 40–550). Signals were identified on the basis of their mass spectra compared with those present in the instrument library (NIST 14). In addition, once the glucidic and organic acid fractions were recognized, proper analytical standards were used in order to confirm the identifications. The semi quantification of all detected gas-chromatographic signals was performed on the basis of the use of two internal standards: turanose for sugar quantification and glutaric acid for organic acid quantification. For each identified compound, the Response Factor (RF) was calculated and the values range between 0.8–1.2.

### 2.6. Characterization of the Volatile Profile

The volatile profile of fermented and unfermented samples was analyzed after 48 h of incubation and after 12 days of storage. Volatiles were characterized by HS-SPME/GC-MS (Head Space-Solid Phase Microextraction/Gas Chromatography-Mass Spectrometry) technique following the protocol reported by Ricci et al. [18]. In brief, 2 mL of cherry juice was placed in a glass vial and added to an aqueous toluene standard solution (0.25 µg/mL). Head space micro-extraction was performed for 30 min at 40 °C after 15 min of equilibration time. A SPME fiber coated with 50/30 µm of Divinylbenzene–Carboxen–Polydimethylsiloxane (DVB/Carboxen/PDMS) was used (Supelco, Bellefonte, PA, USA). The desorption of volatiles was accomplished by exposing the fiber into the GC injector for 2 min at 250 °C. GC–MS analyses were performed on a Thermo Scientific Trace 1300 gas chromatograph coupled to a Thermo Scientific ISQ single quadrupole mass spectrometer equipped with an electronic impact (EI) source. All samples were injected in splitless mode. Helium was used as carrier gas, with a total flow of 1 mL/min. The separation was performed on a SUPELCOWAX 10 capillary column (Supelco, Bellefonte, PA, USA; 30 m × 0.25 mm × 0.25 µm) with the following program gradient: initial temperature, 50 °C for 3 min, linear increase by 5 °C per minute to 200 °C, then maintained for 12 min. The transfer line temperature was 250 °C. The signal acquisition mode was full scan (from 41 m/z to 500 m/z). The main volatile compounds of cherry juices were identified both on the basis of their mass spectra compared with the library NIST 14 mass spectra, as by calculation of linear retention indices (LRI). The semi-quantification of all detected gas-chromatographic signals was performed on the basis of the use of an internal standard (toluene).

### 2.7. Characterization of Polyphenolic Profile of Fermented and Unfermented Cherry Juices

All fermented and unfermented samples were analyzed by an Accela UHPLC 1250 equipped with a linear ion trap-mass spectrometer (MS) (LTQ XL, Thermo Fisher Scientific Inc, San Jose, CA, USA) fitted with a heated-electrospray ionization probe (H-ESI-II, Thermo Fisher Scientific Inc, San Jose, CA, USA). Separation was performed on an Acquity UPLC HSS T3 (2.1 × 100 mm) column coupled with a pre-column Acquity UPLC HSS T3 VanGuard (2.1 × 5 mm) (Waters, Milford, MA, USA). The volume injected was 5 μL, and oven temperature was set to 40 °C. Phenolic profiling was performed following the protocol reported by Ricci et al. [12]. Briefly, the mobile phase was 0.1% (*v/v*) acetonitrile (phase A) and 0.1% (*v/v*) aqueous formic acid (phase B). Elution was performed at a flow rate of 0.3 mL/min. The gradient started with 95% B and 5% A for 0.5 min, then eluent B decreased at 49% and A increased at 51% in 9 min. After 0.5 min, the column was flushed, setting the eluent percentages at 20% B and 80% A for 11.00 min. Finally, the initial conditions were restored (total run time = 17 min). Data processing was performed using Xcalibur 2.2 software from Thermo Fisher Scientific Inc, (San Jose, CA, USA).

### 2.8. Statistical Analysis

To evaluate the normal distribution for each group of independent samples Shapiro-Wilk test was used. One-way ANOVA was applied to discriminate the significant differences among the samples, applying Bonferroni post hoc test and the results were considered different for values of *p* < 0.05. All the detected compounds (volatiles, phenolics, organic acids and sugars) were used as variables for Principal Component Analyses (PCA), which was performed by applying a correlation matrix. All the mentioned analyses were performed on SPSS Statistics 21.0 software (SPSS Inc., Chicago, IL, USA), while hierarchical clustering and heat map were carried out using Heatmapper [21].

## 3. Results

### 3.1. Fermentation and Acidification of Cherry Juice

Cherry juice microflora was checked before fermentation, and no cell viability was observed by plate count. The fermentation was carried out by inoculating four strains of *L. plantarum*, five of *L. rhamnosus*, four of *L. casei* and one of *L. paracasei*. After 48 h of incubation, all *L. plantarum* strains were able to grow in commercial cherry juice (>1 Log cycle). In addition, *L. rhamnosus* 2360 and *L. paracasei* 4186 strains were also able to grow significantly (about 1 Log cycles). However, for most of the tested strains, an increase lower than one half Log cycles or even a decrease was observed. Cell viability remained almost unchanged after 12 days of storage at 4 °C, and only in some cases a slight decrease was observed (Table 2). Lactic acid fermentation and storage did not affect the initial pH = 3.61 ± 0.04 (mean value ± standard deviation). All further analyses were performed considering only the strains that had shown a growth around 1 Log CFU/mL or higher, namely *L. plantarum* 1LE1, POM1, C1, 285, *L. rhamnosus* 2360 and *L. paracasei* 4186 (Table 2). 

### 3.2. Principal Component Analyses: Overview on Volatiles, Phenolic Compounds, Sugars and Organic Acids

**Figure 1:**
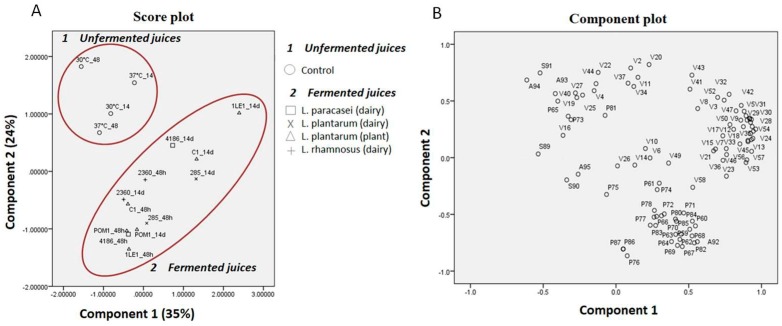
Principal component analyses. (**A**) Score plot obtained from the analysis of unfermented (control) and fermented (*L. plantarum*, *L. rhamnosus* and *L. paracasei*) cherry juice. Each sample is identified by the strain number and the time of incubation. For example, 1LE1_48h is used for strain *L. plantarum* 1LE1 after fermentation. (**B**) Loading plot is based on sugars, organic acids, and volatile and phenolic compounds. Abbreviations are the same as used in Table S1 for organic acids (A) and sugars (S), Table S2 for volatile compounds (V), and Table S4 for polyphenols (P).

The main organic acids (lactic, malic, tartaric and citric) and sugars (fructose, glucose and sucrose) (Table S1) were identified and semi-quantified in fermented and unfermented cherry juice. Among volatile components, alcohols, acids, ketones, esters, terpenes and norisoprenoids were detected and semi-quantified (57 compounds) (Tables S2 and S3A,B). Also, several phenolic acids and flavonoids (Tables S4 and S5) were identified (34 compounds) and quantified (30 compounds). PCA was performed considering the semi-quantified organic acids, sugars and volatiles and the quantified polyphenols, to highlight differences among the analyzed samples. Considering a total of 94 variables, the total variance explained by PCA reached 59%, with component 1 and component 2 describing 35% and 24%, respectively. A defined clustering among fermented and unfermented (control) samples was observed (Figure 1A,B). Unfermented samples were characterized based on those variables showing a negative value on component 1 and a positive value on component 2, such as sucrose, fructose and caffeic acid, at their highest concentrations. On the other hand, fermented juices were all grouped together, even if some differences could be observed. Indeed, after storage, the juices inoculated with 4186, C1, 285 were found to be correlated with slightly positive variables for both components (volatile compounds as propyl acetate) and were clustered separately from the main group. The most different sample was the juice containing *L. plantarum* 1LE1, which was characterized by more positive variables, especially volatile compounds (such as 4-hydroxybutanoic acid, 1-heptanol and trans-geraniol) (Figure 1A,B). 

### 3.3. Sugars and Organic Acid Metabolism

**Figure 2:**
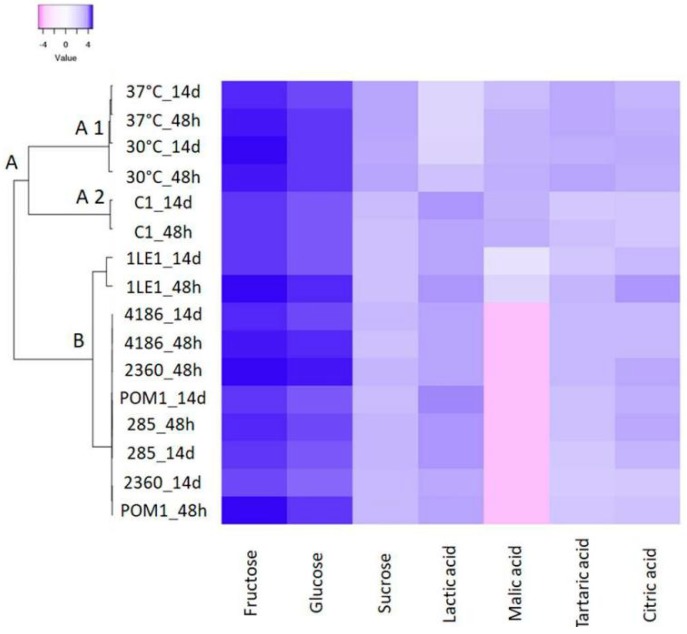
Sugars and organic acid metabolism. Hierarchical clustering and heat map performed on sugars and organic acids detected in fermented juice with *L. plantarum* (C1, 1LE1, POM1 and 285), *L. rhamnosus* (2360) and *L. paracasei* (4186) and unfermented juice (30 °C and 37 °C) after fermentation (48 h) and storage (14 days). A scale ranging from a maximum of 4 (blue) and a minimum of −4 (pink) was used.

Sugar in cherry juice was mainly fructose, glucose and sucrose. Generally, their microbial metabolism was limited (Figure 2, Table S1). As an example, fructose, the most concentrated sugar, was only partially metabolized, especially after storage by *L. plantarum* strains (*p* < 0.05). Microbial fermentation did not change glucose concentration, but it was only metabolized during storage; whereas sucrose significantly decreased in all fermented samples. Lactic acid was the main end product in all fermentations, reaching the highest amount when *L. plantarum* POM1 was used as starter (9.47 ± 0.32 mg/mL). Malolactic fermentation occurred, leading to total degradation of malic acid, in particular when strains of dairy origin were used. Only one strain, *L. plantarum* C1, did not convert malic acid (Figure 2 and Table S1). Tartaric acid metabolism was observed after fermentation and storage, especially by *L. plantarum* strains (*p* < 0.05, reduction of 92%), but it was also observed in *L. rhamnosus* and *L. paracasei* (Figure 2, group A2 and B Table S1). No significant differences were observed for citric acid among fermented and unfermented samples (Figure 2 and Table S1).

### 3.4. Characterization of Volatile Profile

During the characterization of unfermented and fermented cherry juices, carried out using HS-SPME/GC-MS analysis, 57 different compounds were detected in the headspace of every sample. For their identification, the mass spectra were compared with those present in the instrument library NIST 14. Furthermore, linear retention indices (LRIs) were calculated for every signal using the retention time of a linear alkane solution analyzed in the same condition, and the LRIs were compared with those reported in literature. The identification of all detected volatile compounds was reported in Table S2. 

**Figure 3:**
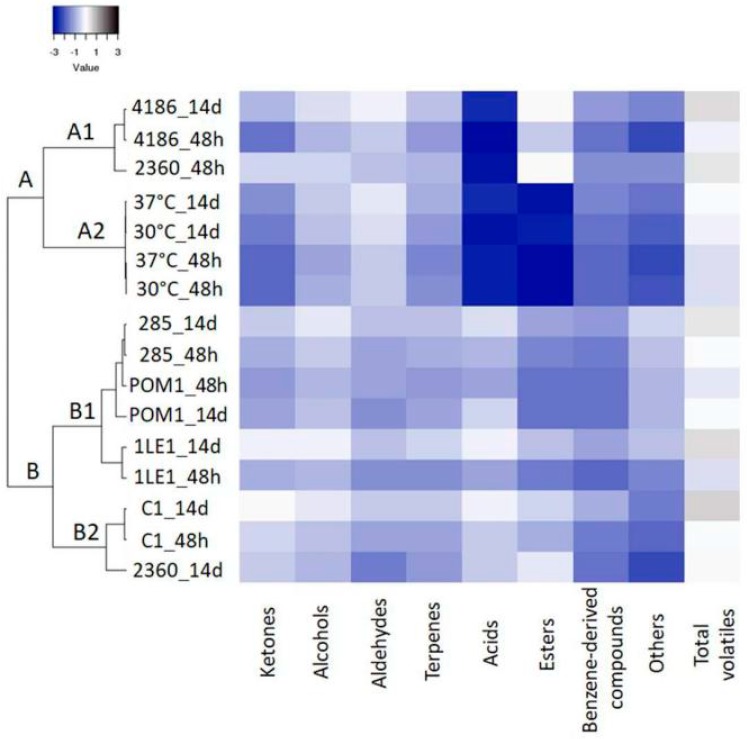
Volatile profile. Hierarchical clustering and heat map performed on sugars and organic acids detected in fermented cherry juice with *L. plantarum* (C1, 1LE1, POM1 and 285), *L. rhamnosus* (2360) and *L. paracasei* (4186) and in unfermented juice (30 °C and 37 °C) after 48 h and 14 days (storage). A scale ranging from a maximum of 3 (black) and a minimum of −3 (blue) was used.

Overall, after fermentation, an increase in the concentration of total volatile compounds was observed. The main identified chemical classes were ketones, alcohols, aldehydes, terpenes, terpenic derivatives and norisoprenoids, acids and esters (Figure 3 and Table S3A,B). In fermented samples, an increased content of ketones was found; among them, acetoin was the most concentrated, especially when strains of plant origin were employed. This compound showed a further increase after storage, reaching the highest concentration when *L. plantarum* C1 was used (Table 3). Benzene methanol was the most abundant alcohol (maximum amount detected with *L. plantarum* 1LE1 after storage, Table 3). Aldehydes were generally not affected by fermentation and storage.

Acids were present at low concentrations both in controls and in fermented juice; an increase after fermentation, and furthermore, at the end of refrigerated storage, was clearly observed, especially when *L. plantarum* strains were employed (Figure 3, group B1 and B2). Acetic acid was the most concentrated compound, especially produced by *L. plantarum*, in particular by the C1 strain (Table 3), but this compound was also produced and converted to the corresponding ester by *L. rhamnosus* and *L. paracasei*; propyl acetate increased after fermentation with *L. rhamnosus* and *L. paracasei* (Table 3).

Overall, after fermentation and refrigerated storage, the concentration of terpenes, terpenic derivatives and norisoprenoids increased in fermented samples, with *β*-linalool being the most abundant one. At the end of storage, when *L. plantarum* (1LE1, 285, C1) and *L. paracasei* 4186 were used as starters, the content of *β*-linalool was significantly increased in comparison to controls. Other volatile compounds derived from the bacterial metabolism were found in fermented cherry juices and, among them, 4-ethylphenol reached its highest concentration when the fermentation was carried out by *L. plantarum* 285, and after storage (Table 3, Figure 3).

### 3.5. Characterization of Polyphenolic Profile

Using LC-MS^n^, 34 polyphenolic compounds were identified, 16 by comparison with authentic standards, while for the others, for which reference compounds were not available, tentative identification was based on the interpretation of their fragmentation patterns obtained from MS^2^ and MS^3^ spectra and by comparison with data reported in literature. Retention times and mass spectral data, along with peak assignments for the identified compounds, are reported in Table S4. Quantified compounds are reported in Table S5. The total polyphenolic concentration did not change upon treatment, but differences inside individual polyphenolic subclasses emerged, suggesting that specific compounds were metabolized by lactic acid bacteria. 

**Figure 4:**
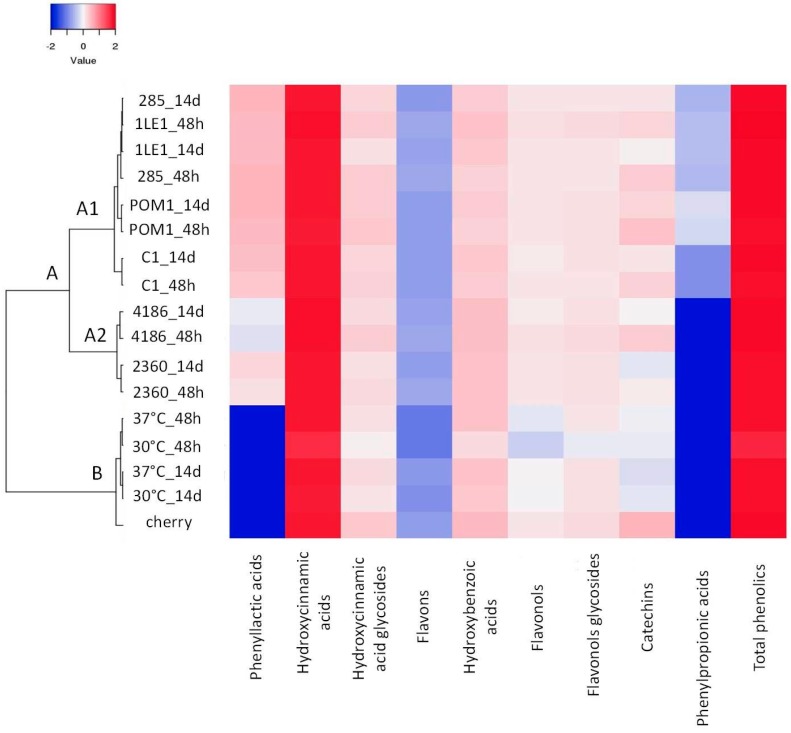
Phenolic compounds. Hierarchical clustering and heat map performed on phenolic compounds detected in fermented juice with *L. plantarum* (C1, 1LE1, POM1 and 285), *L. rhamnosus* (2360) and *L. paracasei* (4186) and unfermented juice (30 °C and 37 °C) after 48 h and 14 days (storage). A scale ranging from a maximum of 2 (red) and a minimum of −2 (blue) was used.

Among hydroxycinnamic acids, caffeic acid and *p*-coumaric acid were mainly affected by fermentation. The conversion of caffeic acid was observed in *L. plantarum* strains, with a complete degradation of the compound by *L. plantarum* 285. The metabolism of caffeic acid led to the accumulation of dihydrocaffeic acid, the only phenylpropionic acid detected (Figure 4, Table 4). *p*-Coumaric acid was totally metabolized by *L. plantarum* 285 and POM1, whereas for the C1 strain a partial conversion was observed after fermentation, and the compound was totally metabolized upon storage (Figure 4, Table 4). Protocatechuic acid was also metabolized by LAB, mainly by *L. plantarum* (Table 4). In addition, all the strains were able to produce *p*-hydroxyphenyllactic acid and phenyllactic acid; the highest levels of these compounds were measured after *L. plantarum* 285 fermentation (1.47 ± 0.16 µg/mL and 2.15 ± 0.15 µg/mL respectively).

## 4. Discussion

*L. plantarum* is the most employed species for lactic acid fermentation of fruit juices, as these strains isolated from plant environments are better adapted to these matrices [10]. Indeed, chemical characteristics of fruits, such as pH and the concentration of sugars and organic acids, make them a hostile environment for microorganisms [22,23,24]. However, a great biodiversity among LAB, species- and strain-dependent, was observed, which allowed them to adopt specific alternative metabolic pathways in adverse conditions using non-conventional carbon sources for the exploitation of alternative substrates or in a global stress response. This adaptation may result in formation of volatile compounds [17,18,23], metabolism of phenolic compounds [11,25], and production of new molecules. Starting from these premises, a large number of dairy strains were used for cherry juice fermentation, and a global view on the metabolism of both dairy and plant-derived microorganisms was proposed.

Cherry juice is recognized to be a stressful substrate for microorganisms, due to its low pH, its high sugar and phenolic content, and the presence of malic acid [23]. This unfavorable environment affects the growth and cell viability of most tested strains, with a particular negative effect on *L. casei* and *L. rhamnosus*, which were not able to grow satisfactorily in this medium. On the contrary, all *L. plantarum* strains, both of dairy and plant origin, showed a good adaptability. Moreover, *L. rhamnosus* 2360 and *L. paracasei* 4186 were also able to ferment cherry juice components and to survive during refrigerated storage, also offering the possibility of conveying viable cells of non-plant origin in this fruit juice. PCA analysis showed a clear separation between unfermented and fermented juices, on account of the significant modulation of the composition of the juice matrix by lactic acid fermentation.

The adaptation to the acid environment of cherry juice is based on the ability of LAB to metabolize malic acid [24], resulting in its almost complete degradation. Actually, all tested dairy and plant strains, independently of species and with the exception of *L. plantarum* C1, completely converted malic acid into lactic acid. Indeed, most LAB can convert malic acid into lactic acid thanks to the malolactic enzyme, decarboxylating malate to lactate by a NAD^+^ and Mn^2+^-dependent malolactic enzyme [23,26,27]. At low pH, the choice of malic acid as the preferred energy source over glucose had already been reported in literature and linked to the increase of intracellular pH and the increase of reducing power [28], with an associated modification of cellular permeability [23]. In the present work, LAB did not consume glucose and fructose during fermentation, whereas sucrose showed a marked decrease. The metabolic activity of the tested strains, especially in *L. rhamnosus* and *L. paracasei,* is mainly based on malolactic fermentation, aimed at the maintenance of vitality, rather than on sugars fermentation, which is mainly correlated with growth [23]. The exploitation of an organic carbon source by a microorganism strongly depends on the type of substrate. Two different works on cherry juice fermentation reported the absence of sugars metabolism in one case [23], and a slight consumption after fermentation in the other [2], the latter being in very good agreement with our results. Observed differences may be related to cultivar, to ripening time, and to the applied processing conditions, which could affect the concentration of nutrients and/or acids [1,29]. In the present work, tartaric acid was also consumed by LAB, especially by *L. plantarum*, in agreement with literature, where *Lactobacillus spp.* were reported to be able to convert it into oxaloacetic acid (and then into lactic acid, acetic acid, and CO_2_), thanks to the tartrate dehydratase enzyme [30,31,32]. Nevertheless, the degradation of tartaric acid is not widespread in LAB, and it has been studied especially in wine. Moreover, not all LAB were able to metabolize tartaric acid in the same way; for instance, differently from *L. plantarum*, in *Lactobacillus brevis*, succinic acid can be produced instead of lactic acid [33]. The acetic acid found in several fermented fruit juices [18,23,34] is characterized by sharp, pungent and vinegar notes. However, thanks to specific bacterial metabolic traits, acetic acid can be converted to the corresponding esters [35,36], such as propyl acetate; this was especially observed after fermentation with *L. rhamnosus* 2360 and at the end of storage using *L. paracasei* 4186. The increase of β-linalool, observed in different fermented samples, could be ascribed to glycosylases produced by LAB, involved in the release of aglycones from glycosylated terpenes [18]. Acetoin was also affected by fermentation and its bio-synthesis can be derived from citrate metabolism [18,27].

Phenolic compounds have been reported to exert health benefits in humans [37], to exhibit antimicrobial activity, and to impact the flavor, taste and color [10] of plant products [38]. Their beneficial activities have been partially correlated with microbial metabolism that can occur during fermentation processes [39]. Moreover, the metabolism of phenolics may contribute to bacterial stress response when microorganisms are in hostile conditions [19,40].

In the present study, hydroxycinnamic acids were transformed by LAB, ideally through phenolic acid decarboxylases or phenolic acid reductases [19]. Caffeic acid was effectively converted into dihydrocaffeic acid, as also already reported in the literature for *L. plantarum* POM1, [11,12,19], with a potential impact on health, due to its more effective capacity to inhibit platelet activation than its phenolic precursor [41] and to its antioxidant effect on endothelial cells [42]. In addition to putative effects on the health-promoting activity of fermentation metabolites, the microbial transformation exerted by LAB may be relevant for other purposes. For example, *p*-coumaric acid was decarboxylated into *p*-vinylphenol and subsequently reduced, possibly by a phenolic acid reductase, to the phenolic volatile 4-ethylphenol, which contributes to aroma in fermented food [19]. The reduction of *p*-vinylphenol was favoured under anaerobic conditions or in absence of electron acceptors, i.e., when fructose is found at high concentration, to increase NAD^+^ quantity [19,43]. Protocatechuic acid was also metabolized [11,12], in particular by *L. plantarum* 285 and POM1, reaching complete depletion, but catechol was not detected. Finally, worthy of note is that phenyllactic acids were produced ex-novo by all the tested strains, probably deriving from amino acid metabolism. Phenylalanine can be converted into phenylpyruvic acid by a transamination reaction, and finally metabolized into phenyllactic acid by hydroxyacid dehydrogenase, while *p*-hydroxyphenyllactic acid may originate from tyrosine metabolism [44]. Their antimicrobial activity against pathogenic strains and moulds has been well documented [44,45], even if the concentrations found in the present study were lower compared to those explaining antimicrobial activity.

## 5. Conclusions

In the present study, an overview on the metabolism of LAB strains, of plant but especially dairy origin, during cherry juice fermentation and further storage, was reported. *L. plantarum* 285, of dairy origin, was the most interesting strain for its ability to modulate the juice phenolic profile, while *L. rhamnosus* 2360 and *L. paracasei* 4186 were useful for modifying and increasing the presence of aromatic compounds. Based on these results, a selection of strains exploitable as starters in cherry juice fermentation can be performed based on sound analytical data. *L. rhamnosus* and *L. paracasei* conferred fruit notes to the juice; on the other hand, *L. plantarum* increased the levels of dihydrocaffeic acid, with putative biological activity, and of phenyllactic acids.

## Figures and Tables

**Table 1:** Microbial strains used in the study and their origin.

Species	Strain	Origin
*L. plantarum*	POM1 *	Tomato (plant)
	C1 *	Carrot (plant)
	1LE1 *	Pineapple (plant)
	285 **	Minas cheese (dairy)
*L. rhamnosus*	2178 **	Parmigiano Reggiano cheese (dairy)
	2140 **	Parmigiano Reggiano cheese (dairy)
	2360 **	Parmigiano Reggiano cheese (dairy)
	1473 **	Parmigiano Reggiano cheese (dairy)
	1019 **	Parmigiano Reggiano cheese (dairy)
*L. casei*	2246 **	Parmigiano Reggiano cheese (dairy)
	2306 **	Parmigiano Reggiano cheese (dairy)
	2057 **	Parmigiano Reggiano cheese (dairy)
	2107 **	Parmigiano Reggiano cheese (dairy)
*L. paracasei*	4186 **	Pecorino cheese (dairy)

* Department of Soil, Plant and Food Science, University of Bari, Italy. ** Department of Food and Drug, University of Parma, Italy.

**Table 2:** Lactic acid bacteria growth. Difference in Log CFU/mL detected between cells number after 48 h of incubation and the initial value, Δ(T_48h_–T_0_), and between cells number after storage (14 days) and the initial value, Δ(T_14d_–T_0_). The isolation origin (plant/dairy products) is specified. Only the strains able to grow are reported.

Species	Strain	Origin	Δ(T_48h_–T_0_)	Δ(T_14d_–T_0_)
*L. plantarum*	POM1	Plant	1.82 ± 0.05	1.69 ± 0.03
	C1	Plant	1.22 ± 0.10	1.11 ± 0.04
	1LE1	Plant	1.32 ± 0.07	1.39 ± 0.17
	285	Dairy	1.44 ± 0.07	1.46 ± 0.03
*L. rhamnosus*	2360	Dairy	1.02 ± 0.10	0.97 ± 0.03
*L. paracasei*	4186	Dairy	0.96 ± 0.13	0.80 ± 0.14

**Table 3:** Volatile compounds. Volatile compound concentrations (ng/mL) in fermented and unfermented cherry juice.

**48 Hours**								
**Compound**	**37 °C**	**2360**	**4186**	**30 °C**	**1LE1**	**285**	**C1**	**POM1**
acetoin	0.001 ± 0.000	260.679 ± 34.426 *	5.909 ± 0.480	0.002 ± 0.000	71.373 ± 4.486 *	76.300 ± 18.884 *	287.902 ± 14.971*	44.022 ± 1.515 *
benzene methanol	51.130 ± 13.431	164.411 ± 4.744 *	70.560 ± 13.868	64.548 ± 0.700	70.513 ± 1.502	158.829 ± 3.660 *	95.692 ± 15.202	90.078 ± 11.908 *
β-linalool	13.742 ± 3.515	39.630 ± 2.624 *	21.464 ± 2.627 *	15.670 ± 0.326	15.305 ± 0.181	28.975 ± 0.424 *	19.964 ± 2.326 *	19.593 ± 1.604 *
acetic acid	0.087 ± 0.030	0.017 ± 0.017 *	0.014 ± 0.002 *	0.013 ± 0.011	54.831 ± 18.642 *	125.405 ± 10.324 *	184.836 ± 21.933 *	69.201 ± 9.371 *
propyl acetate	0.009 ± 0.005	1186.731 ± 460.827 *	201.607 ± 26.688	0.008 ± 0.005	24.680 ± 0.422 *	27.876 ± 5.804 *	83.370 ± 6.871 *	18.539 ± 0.273 *
4-ethylphenol	0.341 ± 0.016	29.025 ± 12.443 *	1.828 ± 0.232	0.503 ± 0.144	23.832 ± 4.294 *	150.460 ± 3.007 *	8.997 ± 3.734 *	106.967 ± 6.068 *
**14 Days**								
**Compound**	**37 °C**	**2360**	**4186**	**30 °C**	**1LE1**	**285**	**C1**	**POM1**
acetoin	0.004 ± 0.005	180.078 ± 22.938 *	65.013 ± 1.293 *	0.004 ± 0.000	580.569 ± 61.646 *	161.905 ± 50.669	1058.883 ± 222.855 *	63.563 ± 22.691
benzene methanol	146.075 ± 6.008	79.259 ± 21.526 *	250.467 ± 32.932 *	99.225 ± 1.022	462.867 ± 76.120 *	311.721 ± 47.740 *	327.123 ± 54.469 *	116.204 ± 42.234
β-linalool	41.464 ± 3.599	19.467 ± 2.875 *	70.918 ± 15.120 *	27.205 ± 2.210	92.675 ± 20.477 *	57.517 ± 9.385 *	68.307 ± 4.195 *	22.431 ± 8.860
acetic acid	0.024 ± 0.009	228.633 ± 71.471 *	0.354 ± 0.192	0.013 ± 0.009	535.465 ± 92.920 *	305.391 ± 5.248 *	737.615 ± 193.995 *	257.568 ± 75.381 *
propyl acetate	0.026 ± 0.004	509.194 ± 12.345 *	1230.067 ± 222.171 *	0.015 ± 0.014	160.882 ± 2.895 *	61.035 ± 14.937 *	281.546 ± 28.411 *	16.706 ± 5.548
4-ethylphenol	0.848 ± 0.141	3.210 ± 0.751	21.854 ± 5.943 *	0.608 ± 0.144	129.556 ± 2.648 *	281.248 ± 21.113 *	12.287 ± 0.017 *	136.453 ± 43.612 *

* significant differences between the concentrations of each compound observed in fermented cherry juices and in the respective control; 37 °C for *L. rhamnosus* 2360 and *L. paracasei* 4186, 30 °C for *L. plantarum* 1LE1, 285, C1 and POM1.

**Table 4:** Phenolic compounds. Phenolic compounds (µg/mL) metabolized by lactic acid bacteria during cherry juice fermentation and further storage.

**48 Hours**								
**Compounds**	**37 °C**	**2360**	**4186**	**30 °C**	**1LE1**	**285**	**C1**	**POM1**
*p*-Hydroxyphenyllactic acid	ND	0.986 ± 0.121 *	0.209 ± 0.047 *	ND	1.229 ± 0.187 *	1.462 ± 0.089 *	1.241 ± 0.148 *	1.134 ± 0.125 *
Phenyllactic acid	ND	0.483 ± 0.062 *	0.449 ± 0.024 *	ND	2.098 ± 0.187 *	1.905 ± 0.150 *	1.219 ± 0.076 *	1.930 ± 0.026 *
Caffeic acid	0.633 ± 0.025	0.743 ± 0.038 *	0.722 ± 0.030 *	0.586 ± 0.033	0.406 ± 0.270	ND	0.305 ± 0.040	0.062 ± 0.014 *
*p*-Coumaric acid	0.609 ± 0.274	0.594 ± 0.061	0.486 ± 0.105	0.288 ± 0.124	0.484 ± 0.134	ND	0.354 ± 0.207	ND
Protocatechuic acid	0.457 ± 0.197	0.508 ± 0.023	0.382 ± 0.103	0.253 ± 0.114	0.380 ± 0.131	ND	0.308 ± 0.273	ND
Dihydrocaffeic acid	ND	ND	ND	ND	0.307 ± 0.222	0.284 ± 0.043	0.131 ± 0.062	0.568 ± 0.070 *
**14 Days**								
**Compounds**	**37 °C**	**2360**	**4186**	**30 °C**	**1LE1**	**285**	**C1**	**POM1**
*p*-Hydroxyphenyllactic acid	ND	1.272 ± 0.159 *	0.259 ± 0.030 *	ND	1.225 ± 0.025 *	1.473 ± 0.158 *	1.271 ± 0.115 *	1.295 ± 0.131 *
Phenyllactic acid	ND	0.559 ± 0.034 *	0.513 ± 0.006 *	ND	1.986 ± 0.087 *	2.150 ± 0.153 *	1.494 ± 0.141 *	2.208 ± 0.095 *
Caffeic acid	0.685 ± 0.062	0.789 ± 0.050	0.668 ± 0.038	0.702 ± 0.037	0.345 ± 0.241 *	ND	0.259 ± 0.024 *	0.092 ± 0.020 *
*p*-Coumaric acid	0.670 ± 0.294	0.472 ± 0.050	0.485 ± 0.126	0.412 ± 0.103	0.337 ± 0.019	ND	ND	ND
Protocatechuic acid	0.560 ± 0.290	0.484 ± 0.170	0.497 ± 0.217	0.357 ± 0.148	0.285 ± 0.052	ND	0.459 ± 0.247	ND
Dihydrocaffeic acid	ND	ND	ND	ND	0.317 ± 0.314	0.278 ± 0.022	0.124 ± 0.040	0.609 ± 0.041 *

* significant differences between the concentrations of each compound observed in fermented cherry juices and in the respective control, 37 °C for *L. rhamnosus* 2360 and *L. paracasei* 4186, 30 °C for *L. plantarum* 1LE1, 285, C1 and POM1. ND: not detected.

## Supplementary Materials

The following are available online at http://www.mdpi.com/2072-6643/11/2/213/s1, Table S1. Carbohydrates and organic acids of fermented and unfermented cherry juice. Concentration (µg/mL) of carbohydrates (fructose, glucose and sucrose) and organic acids (lactic acid, malic acid, tartaric acid, citric acid) detected in fermented cherry juices (1LE1, 285, C1, POM1, 4186 and 2360) and in unfermented cherry juices used as controls (30 °C and 37 °C). All the samples were treated in the same conditions and the analyses were carried out after 48 h of incubation and after storage (14 days). For each compound, a specific variable, used in PCA analyses, was assigned. Table S2. Identification of volatile compounds found in sweet cherry juices fermented with *L. plantarum* (POM1, C1, 1LE1, 285), *L. rhamnosus* (2360), *L. paracasei* (4186) and in controls (juices treated at 30 °C and 37 °C). MS: mass spectrometer; LRI: linear retention index. A specific variable, used in PCA analyses, was assigned for each compound. Table S3A. Concentration (ng/mL) of volatile compounds identified in sweet cherry juices fermented with *L. plantarum* (POM1, C1, 1LE1, 285), *L. rhamnosus* (2360), *L. paracasei* (4186) and in controls (juices treated at 30 °C and 37 °C) after 48 h. Table S3B. Concentration (ng/mL) of volatile compounds identified in sweet cherry juices fermented with *L. plantarum* (POM1, C1, 1LE1, 285), *L. rhamnosus* (2360), *L. paracasei* (4186) and in controls (juices treated at 30 °C and 37 °C) after storage (14 days). Table S4. Identification of phenolic compounds. Chromatographic and mass spectral characteristics of phenolic compounds detected in started and unstarted elderberry juices. For quantified compounds a specific variable, used in PCA analyses, was assigned. Table S5. Phenolic profile. Concentration (µg/mL) of phenolic compounds detected in unfermented (37 °C and 30 °C) and fermented with *L. rhamnosus* 2360, *L. paracasei* 4186, *L. plantarum* 1LE1, 285, C1 and POM1 cherry juice after 48 h of fermentation and further storage (14 days).
