# Supplementary material for: Use of Dairy and Plant-Derived Lactobacilli as Starters for Cherry Juice Fermentation
Source: Nutrients. 2019 Jan 22;11(2):213. doi: 10.3390/nu11020213 (PMC6412669; doi:10.3390/nu11020213)
Supplement: Supplementary file 1 [file nutrients-11-00213-s001.zip › Supplememntary materials/Supplementary table S1.docx]

Table S1. **Carbohydrates and organic acids of fermented and unfermented cherry juice.** Concentration (mg/mL) of carbohydrates (fructose, glucose and sucrose) and organic acids (lactic acid, malic acid, tartaric acid, citric acid) detected in fermented cherry juices (1LE1, 285, C1, POM1, 4186 and 2360) and in unfermented cherry juices used as controls (30°C and 37°C). All the samples were treated in the same conditions and the analyses were carried out after 48 hours of incubation and after storage (14 days). For each compound a specific variable, used in PCA analyses, was assigned.

|  |  | 48 Hours | | | | | | | | | | | | | | | | | | | | | | | | | | | | | | |
| --- | --- | --- | --- | --- | --- | --- | --- | --- | --- | --- | --- | --- | --- | --- | --- | --- | --- | --- | --- | --- | --- | --- | --- | --- | --- | --- | --- | --- | --- | --- | --- | --- |
|  |  | 30°C | | |  | 1LE1 | | |  | 285 | | |  | C1 | | |  | POM1 | | |  | 37°C | | |  | 4186 | | |  | 2360 | | |
| S89 | Fructose | 45.74 | ± | 4.34 |  | 54.32 | ± | 26.55 |  | 38.87 | ± | 0.67 |  | 28.37 | ± | 5.19 |  | 54.20 | ± | 5.06 |  | 49.56 | ± | 0.58 |  | 44.34 | ± | 7.09 |  | 54.84 | ± | 1.21 |
| S90 | Glucose | 27.02 | ± | 2.70 |  | 39.50 | ± | 2.54 |  | 23.32 | ± | 1.11 |  | 18.87 | ± | 3.069 |  | 31.22 | ± | 3.36 |  | 27.82 | ± | 4.53 |  | 33.47 | ± | 2.91 |  | 40.57 | ± | 2.89 |
| S91 | Sucrose | 5.84 | ± | 4.22 |  | 0.99 | ± | 0.14* |  | 2.06 | ± | 0.90* |  | 1.13 | ± | 0.18* |  | 1.51 | ± | 0.16* |  | 6.18 | ± | 0.57 |  | 1.17 | ± | 0.20* |  | 1.98 | ± | 0.13* |
| A92 | Lactic acid | 0.81 | ± | 0.09 |  | 7.75 | ± | 2.54* |  | 8.44 | ± | 0.16* |  | 5.75 | ± | 1.98* |  | 6.58 | ± | 2.45* |  | 0.15 | ± | 0.04 |  | 5.65 | ± | 0.53* |  | 5.99 | ± | 0.59* |
| A93 | Malic acid | 3.23 | ± | 0.91 |  | 0.13 | ± | 0.14 |  | ND | | |  | 2.95 | ± | 0.69 |  | ND | | |  | 2.59 | ± | 0.14 |  | ND | | |  | ND | | |
| A94 | Tartaric acid | 6.67 | ± | 0.66 |  | 2.19 | ± | 0.93 |  | 1.09 | ± | 0.86* |  | 0.95 | ± | 0.17* |  | 0.61 | ± | 0.04* |  | 4.51 | ± | 0.67 |  | 1.54 | ± | 0.64 |  | 1.61 | ± | 0.26* |
| A95 | Citric acid | 3.37 | ± | 1.08 |  | 8.28 | ± | 0.93 |  | 5.42 | ± | 0.22 |  | 0.63 | ± | 0.36 |  | 0.81 | ± | 0.44 |  | 3.23 | ± | 1.62 |  | 1.48 | ± | 0.06 |  | 4.99 | ± | 2.53 |
|  |  |  |  |  |  |  |  |  |  |  |  |  |  |  |  |  |  |  |  |  |  |  |  |  |  |  |  |  |  |  |  |  |
|  |  | 14 Days | | | | | | | | | | | | | | | | | | | | | | | | | | | | | | |
|  |  | 30°C | | |  | 1LE1 | | |  | 285 | | |  | C1 | | |  | POM1 | | |  | 37°C | | |  | 4186 | | |  | 2360 | | |
| S89 | Fructose | 62.47 | ± | 10.34 |  | 27.42 | ± | 3.02* |  | 29.87 | ± | 5.70* |  | 28.92 | ± | 3.93* |  | 30.68 | ± | 2.74* |  | 38.84 | ± | 8.05 |  | 34.43 | ± | 2.03 |  | 22.71 | ± | 2.33* |
| S90 | Glucose | 30.35 | ± | 7.39 |  | 20.48 | ± | 1.40* |  | 19.97 | ± | 5.05* |  | 20.40 | ± | 0.53* |  | 19.47 | ± | 2.61* |  | 22.10 | ± | 0.21 |  | 22.31 | ± | 2.68 |  | 15.50 | ± | 2.44* |
| S91 | Sucrose | 4.59 | ± | 1.44 |  | 1.15 | ± | 0.05* |  | 2.19 | ± | 0.38* |  | 1.43 | ± | 0.08* |  | 1.42 | ± | 0.00* |  | 6.18 | ± | 0.57 |  | 1.51 | ± | 0.73* |  | 1.72 | ± | 0.47* |
| A92 | Lactic acid | 0.19 | ± | 0.09 |  | 6.73 | ± | 0.17* |  | 7.92 | ± | 1.78* |  | 7.90 | ± | 2.04* |  | 9.47 | ± | 0.31* |  | 0.15 | ± | 0.36 |  | 6.04 | ± | 0.68* |  | 5.54 | ± | 0.42* |
| A93 | Malic acid | 2.36 | ± | 0.14 |  | 0.04 | ± | 0.03 |  | ND | | |  | 2.38 | ± | 0.66 |  | ND | | |  | 1.43 | ± | 0.12 |  | ND | | |  | ND | | |
| A94 | Tartaric acid | 3.18 | ± | 0.04 |  | 0.73 | ± | 0.03* |  | 0.56 | ± | 0.01* |  | 0.61 | ± | 0.04* |  | 0.94 | ± | 0.05* |  | 4.52 | ± | 0.67 |  | 1.54 | ± | 0.64* |  | 0.40 | ± | 0.07* |
| A95 | Citric acid | 4.28 | ± | 0.09 |  | 1.52 | ± | 0.07 |  | 2.12 | ± | 0.34 |  | 0.76 | ± | 0.12 |  | 3.01 | ± | 0.04 |  | 2.24 | ± | 0.82 |  | 1.78 | ± | 0.46 |  | 0.55 | ± | 0.10 |

ND not detected

* significant differences between the concentrations of each compound observed in fermented cherry juices and in the respective control, 37°C for *L. rhamnosus* 2360 and *L. paracasei* 4186, 30°C for *L. plantarum* 1LE1, 285, C1 and POM1.
