# Supplementary material for: Use of Dairy and Plant-Derived Lactobacilli as Starters for Cherry Juice Fermentation
Source: Nutrients. 2019 Jan 22;11(2):213. doi: 10.3390/nu11020213 (PMC6412669; doi:10.3390/nu11020213)
Supplement: Supplementary file 1 [file nutrients-11-00213-s001.zip › Supplememntary materials/supplementary table S2.docx]

Table S2. **Identification of volatile compounds.** Identification of volatile compounds found in sweet cherry juices fermented with *L. plantarum* (POM1*,* C1*,* 1LE1*,* 285), *L. rhamnosus* (2360), *L. paracasei* (4186) and in controls (juices treated at 30°C and 37°C). MS: mass spectrometer; LRI: linear retention index. A specific variable, used in PCA analyses, was assigned for each compound.

| Peak number | Identification | Aromatic note | LRI | Identification method | References |
| --- | --- | --- | --- | --- | --- |
| V1 | 2-Ethoxy-2-methyl-propane |  | 717 | MS |  |
| V2 | Acetone | Ethereal, apple, pear | 810 | MS |  |
| V3 | Isovaleraldehyde | Ethereal, aldehydic | 888 | MS + LRI | Qian & Reineccius, 2003 |
| V4 | Ethanol | Strong, alcoholic | 902 | MS + LRI | Goodner, 2008 |
| V5 | Propyl acetate | Estery, fruity, ethereal, tutti frutti, banana, honey | 977 | MS + LRI | Kevei & Kozma, 1976 |
| V6 | Ethyl benzene |  | 1117 | MS + LRI | Alasalvar et al., 2005 |
| V7 | Unidentified terpene |  | 1133 | MS |  |
| V8 | Limonene | Citrus | 1175 | MS + LRI | Cirlini et al., 2012 |
| V9 | 2-Heptanone | Cheesy, fruity, coconut, waxy, green | 1180 | MS + LRI | Tatsuka et al., 1990 |
| V10 | 4-Methyl-2-heptanone |  | 1206 | MS + LRI | Canuti et al., 2009 |
| V11 | 2-Pentylfuran | Green, waxy, cooked, caramellic | 1218 | MS + LRI | Mahadevan & Farmer, 2006 |
| V12 | Isopentyl alcohol | Fermented, fruity, banana, ethereal, cognac | 1222 | MS + LRI | Dall’Asta et al., 2011 |
| V13 | Ethyl isoamyl ketone |  | 1249 | MS |  |
| V14 | Styrene | Sweet, balsamic, floral | 1252 | MS + LRI | Bianchi et al., 2007 |
| V15 | 3-Pentenol |  | 1256 | MS |  |
| V16 | m-Cymene |  | 1257 | MS + LRI | Cavalli et al., 2003b |
| V17 | Isobutenylcarbinol |  | 1261 | MS + LRI | Wirth et al., 2001 |
| V18 | Octanal | Aldehydic, green, citrus | 1285 | MS + LRI | Bianchi et al., 2007 |
| V19 | 1-Decen-3-one |  | 1298 | MS |  |
| V20 | Acetoin | Sweet, buttery, creamy, dairy | 1302 | MS + LRI | Bianchi et al., 2007 |
| V21 | 2-Methyl-2-octanal | Floral, rose, lily, muguet | 1319 | MS |  |
| V22 | Prenol | Green, fruity | 1325 | MS + LRI | Lee et al., 2005 |
| V23 | Hexanol | Green, fruity, apple skin, oily | 1353 | MS + LRI | Cirlini et al., 2012 |
| V24 | Dimethyl trisulfide | Sulfureous, alliaceous, savory, meaty, fresh, vegetable | 1376 | MS + LRI | Pozo-Bayon et al., 2007 |
| V25 | 2-Nonanone | Green, fruity, cheesy, buttery, dairy | 1385 | MS + LRI | Fernandez-Segovia et al., 2006 |
| V26 | Nonanal | Aldehydic, citrus, cucumber, melon, raw potato, oily, coconut, nutty | 1390 | MS + LRI | Mahmood et al., 2004 |
| V27 | (E)-2-Hexen-1-ol | Fruity, green, leafy | 1406 | MS + LRI | Bianchi et al. 2007 |
| V28 | m-Di-tert-butylbenzene |  | 1421 | MS |  |
| V29 | α-Ionene |  | 1440 | MS |  |
| V30 | trans-Linalool dioxide |  | 1441 | MS |  |
| V31 | Amyl vinyl carbinol | Mushroom, earthy, green, oily, vegetable, umami, savory, brothy | 1449 | MS + LRI | Pinto et al., 2006 |
| V32 | 1-Heptanol | Fermented, oily, nutty, fatty, green, aldehydic | 1454 | MS + LRI | Cirlini et al., 2012 |
| V33 | Furfural | Sweet, woody bready, nutty, caramellic, astringent | 1469 | MS + LRI | Pozo-Bayon et al., 2007 |
| V34 | 2-Ethyl-1-hexanol | Sweet, fatty, fruity | 1488 | MS + LRI | Mahattanatawee et al., 2007 |
| V35 | Acetic acid | Pungent, sharp, vinegar | 1488 | MS + LRI | Bianchi et al., 2007 |
| V36 | Benzaldehyde | Sweet, oily, almond cherry, nutty, woody | 1526 | MS + LRI | Bianchi et al., 2007 |
| V37 | β-Linalool | Floral | 1547 | MS + LRI | Cirlini et al., 2012 |
| V38 | Octanol | Waxy, green, orange | 1555 | MS + LRI | Dall’Asta et al., 2011 |
| V39 | Pivalic acid |  | 1584 | MS + LRI | Johanningsmeier & McFeeters , 2011 |
| V40 | 4-Methyldihydro-2(3H)-furanone |  | 1621 | MS + LRI | Cho et al., 2006 |
| V41 | 4-Hydroxybutanoic acid |  | 1635 | MS |  |
| V42 | Safranal | Woody, phenolic spicy, fruity, herbal | 1646 | MS + LRI | Kaypak & Avsar, 2008 |
| V43 | p-Tolualdehyde | Fruity, cherry, phenolic | 1651 | MS + LRI | Miles et al., 1965 |
| V44 | 1-Nonanol | Fresh, fatty, floral | 1657 | MS + LRI | Dall’Asta et al., 2011 |
| V45 | 2-Furanmethanol | Sweet, caramellic | 1664 | MS + LRI | Dall’Asta et al., 2011 |
| V46 | α,4-Dimethyl-3-cyclohexene-1-acetaldehyde | Spicy, herbal | 1687 | MS + LRI | Garneau et al., 1994 |
| V47 | p-Menthen-8-ol |  | 1696 | MS |  |
| V48 | Naphthalene | Pungent, dry | 1740 | MS + LRI | Alasalvar et al., 2005 |
| V49 | 1-1-6-Trimethyl-1,2-dihydronaphthalene (TDN) |  | 1742 | MS + LRI | López et al., 2004 |
| V50 | Decanol | Aldehydic, waxy, green, fatty, tart, floral | 1759 | MS + LRI | Riu-Aumatell et al., 2005 |
| V51 | Methyl salicylate | Wintergreen, mint | 1779 | MS + LRI | Kaack, 2005 |
| V52 | trans-Geraniol | Sweet, floral, fruity, rose | 1846 | MS + LRI | Goodner, 2008 |
| V53 | Benzene methanol | Fruity, balsamic | 1879 | MS + LRI | Nagarajan et al., 2001 |
| V54 | Benzene ethanol | Floral, sweet, rose, bready | 1914 | MS + LRI | Botelho et al., 2007 |
| V55 | p-Mentha-1(7), 8(10)-dien-9-ol |  | 1996 | MS |  |
| V56 | Eugenol | Sweet, warm, spicy, clove, woody | 2157 | MS + LRI | Goodner, 2008 |
| V57 | 4-Ethylphenol | Phenolic | 2165 | MS + LRI | Ferreira et al., 2001 |
