# Supplementary material for: Use of Dairy and Plant-Derived Lactobacilli as Starters for Cherry Juice Fermentation
Source: Nutrients. 2019 Jan 22;11(2):213. doi: 10.3390/nu11020213 (PMC6412669; doi:10.3390/nu11020213)
Supplement: Supplementary file 1 [file nutrients-11-00213-s001.zip › Supplememntary materials/Supplementary table S3A and S3B.docx]

Table S3A. **Volatile compounds.** Concentration (ng/mL) of volatile compounds identified in sweet cherry juices fermented with L. plantarum (POM1, C1, 1LE1, 285), L. rhamnosus (2360), L. paracasei (4186) and in controls (juices treated at 30°C and 37°C) after 48 hours.

| Compound | | 37 °C | | | 2360 | | | 4186 | | | 30 °C | | | 1LE1 | | | 285 | | | C1 | | | POM1 | | | |
| --- | --- | --- | --- | --- | --- | --- | --- | --- | --- | --- | --- | --- | --- | --- | --- | --- | --- | --- | --- | --- | --- | --- | --- | --- | --- | --- |
|  |  | | | | | | | | | | | | | | | | | | | | | | | | | |
| ***Ketones*** | | | | | | | | | | | | | | | | | | | | | | | | | | |
| acetone | | 5.340 | ± | 0.256 | 13.997 | ± | 1.524 | 4.883 | ± | 0.137 | 4.819 | ± | 0.034 | 3.500 | ± | 0.042 | 7.026 | ± | 0.908 | 3.254 | ± | 0.339 | 4.029 | ± | 0.084 |  |
| 2-heptanone | | 0.331 | ± | 0.467 | 2.598 | ± | 0.295 | 0.183 | ± | 0.223 | 0.461 | ± | 0.436 | 0.015 | ± | 0.012 | 1.172 | ± | 0.129 | 0.509 | ± | 0.070 | 0.174 | ± | 0.243 |  |
| 4-methyl-2-heptanone | | 1.216 | ± | 0.318 | 0.014 | ± | 0.008 | 0.007 | ± | 0.006 | 1.469 | ± | 0.127 | 0.408 | ± | 0.125 | 0.746 | ± | 0.042 | 0.492 | ± | 0.034 | 0.007 | ± | 0.002 |  |
| ethyl isoamyl ketone | | 0.003 | ± | 0.003 | 1.215 | ± | 0.172 | 0.193 | ± | 0.081 | 0.004 | ± | 0.003 | 0.069 | ± | 0.007 | 0.221 | ± | 0.002 | 0.137 | ± | 0.023 | 0.017 | ± | 0.013 |  |
| 1-decen-3-one | | 0.263 | ± | 0.049 | 0.003 | ± | 0.001 | 0.077 | ± | 0.107 | 0.164 | ± | 0.127 | 0.000 | ± | 0.001 | 0.014 | ± | 0.015 | 0.041 | ± | 0.041 | 0.008 | ± | 0.004 |  |
| acetoin | | 0.001 | ± | 0.000 | 260.679 | ± | 34.426 | 5.909 | ± | 0.480 | 0.002 | ± | 0.000 | 71.373 | ± | 4.486 | 76.300 | ± | 18.884 | 287.902 | ± | 14.971 | 44.022 | ± | 1.515 |  |
| 2-nonanone | | 0.004 | ± | 0.003 | 6.596 | ± | 0.274 | 0.013 | ± | 0.013 | 0.003 | ± | 0.001 | 0.015 | ± | 0.003 | 0.225 | ± | 0.081 | 0.018 | ± | 0.006 | 0.014 | ± | 0.001 |  |
| *Total* | | 7.159 | ± | 1.096 | 285.103 | ± | 36.134 | 11.264 | ± | 0.361 | 6.922 | ± | 0.720 | 75.380 | ± | 4.316 | 85.704 | ± | 19.803 | 292.353 | ± | 15.438 | 48.271 | ± | 1.859 |  |
|  |  | | | | | | | | | | | | | | | | | | | | | | | | | |
| ***Alcohols*** | | | | | | | | | | | | | | | | | | | | | | | | | | |
| ethanol | | 3.156 | ± | 0.391 | 8.538 | ± | 1.349 | 4.133 | ± | 0.325 | 3.723 | ± | 0.119 | 3.029 | ± | 0.083 | 5.866 | ± | 0.619 | 4.071 | ± | 0.326 | 4.484 | ± | 0.508 |  |
| isopentyl alcohol | | 0.179 | ± | 0.070 | 1.681 | ± | 0.508 | 0.170 | ± | 0.075 | 0.003 | ± | 0.000 | 0.821 | ± | 0.213 | 1.153 | ± | 0.011 | 0.586 | ± | 0.145 | 0.664 | ± | 0.554 |  |
| 3-pentenol | | 0.092 | ± | 0.128 | 0.006 | ± | 0.003 | 0.004 | ± | 0.001 | 0.356 | ± | 0.502 | 0.005 | ± | 0.001 | 0.002 | ± | 0.002 | 0.002 | ± | 0.001 | 0.827 | ± | 1.167 |  |
| isobutenylcarbinol | | 0.359 | ± | 0.185 | 3.148 | ± | 0.453 | 0.506 | ± | 0.712 | 0.004 | ± | 0.000 | 1.317 | ± | 0.738 | 2.579 | ± | 0.487 | 0.005 | ± | 0.003 | 2.094 | ± | 0.622 |  |
| hexanol | | 0.704 | ± | 0.127 | 2.098 | ± | 0.424 | 1.120 | ± | 0.056 | 0.816 | ± | 0.084 | 1.875 | ± | 0.168 | 3.497 | ± | 0.174 | 2.235 | ± | 0.248 | 2.309 | ± | 0.208 |  |
| (E)-2-hexen-1-ol | | 10.468 | ± | 1.595 | 25.830 | ± | 2.156 | 12.885 | ± | 0.371 | 12.479 | ± | 0.040 | 11.673 | ± | 0.282 | 22.592 | ± | 0.628 | 13.236 | ± | 2.822 | 17.685 | ± | 1.788 |  |
| amyl vinyl carbinol | | 0.496 | ± | 0.189 | 0.633 | ± | 0.162 | 0.187 | ± | 0.169 | 0.495 | ± | 0.180 | 0.446 | ± | 0.340 | 0.705 | ± | 0.031 | 0.358 | ± | 0.069 | 0.239 | ± | 0.006 |  |
| 1-heptanol | | 0.068 | ± | 0.022 | 0.067 | ± | 0.040 | 0.119 | ± | 0.126 | 0.089 | ± | 0.021 | 0.528 | ± | 0.032 | 0.882 | ± | 0.122 | 0.658 | ± | 0.007 | 0.375 | ± | 0.254 |  |
| 2-ethyl-1-hexanol | | 1.629 | ± | 0.462 | 5.359 | ± | 1.204 | 2.872 | ± | 0.480 | 2.056 | ± | 0.133 | 1.873 | ± | 0.193 | 3.917 | ± | 0.433 | 7.281 | ± | 3.477 | 2.910 | ± | 0.777 |  |
| octanol | | 3.364 | ± | 0.791 | 8.419 | ± | 0.975 | 5.200 | ± | 1.363 | 3.596 | ± | 0.463 | 4.465 | ± | 0.210 | 7.831 | ± | 0.570 | 5.716 | ± | 0.462 | 4.813 | ± | 1.201 |  |
| 1-nonanol | | 0.037 | ± | 0.036 | 0.691 | ± | 0.071 | 2.170 | ± | 0.880 | 0.039 | ± | 0.014 | 2.828 | ± | 0.215 | 4.945 | ± | 0.439 | 4.492 | ± | 0.249 | 1.237 | ± | 0.467 |  |
| 2-furanmethanol | | 0.454 | ± | 0.063 | 11.002 | ± | 0.123 | 1.011 | ± | 0.197 | 0.625 | ± | 0.091 | 1.841 | ± | 0.274 | 5.907 | ± | 0.357 | 7.694 | ± | 0.660 | 2.410 | ± | 0.503 |  |
| decanol | | 1.503 | ± | 0.545 | 5.067 | ± | 0.220 | 2.938 | ± | 1.097 | 1.873 | ± | 0.066 | 3.231 | ± | 0.078 | 7.269 | ± | 0.351 | 5.855 | ± | 0.340 | 2.755 | ± | 0.726 |  |
| benzene methanol | | 51.130 | ± | 13.431 | 164.411 | ± | 4.744 | 70.560 | ± | 13.868 | 64.548 | ± | 0.700 | 70.513 | ± | 1.502 | 158.829 | ± | 3.660 | 95.692 | ± | 15.202 | 90.078 | ± | 11.908 |  |
| benzene ethanol | | 2.911 | ± | 0.604 | 8.214 | ± | 0.853 | 2.757 | ± | 0.699 | 4.529 | ± | 0.055 | 4.598 | ± | 0.919 | 11.132 | ± | 0.126 | 6.623 | ± | 1.644 | 5.263 | ± | 0.190 |  |
| *Total* | | 76.551 | ± | 18.011 | 245.163 | ± | 10.997 | 106.632 | ± | 19.655 | 95.230 | ± | 0.480 | 109.044 | ± | 4.535 | 237.105 | ± | 2.060 | 154.504 | ± | 25.003 | 138.144 | ± | 16.181 |  |
|  |  | | | | | | | | | | | | | | | | | | | | | | | | | |
| ***Aldehydes*** | | | | | | | | | | | | | | | | | | | | | | | | | | |
| isovaleraldehyde | | 0.934 | ± | 0.149 | 0.045 | ± | 0.017 | 0.262 | ± | 0.047 | 0.647 | ± | 0.107 | 0.020 | ± | 0.012 | 0.024 | ± | 0.011 | 0.018 | ± | 0.007 | 0.029 | ± | 0.002 |  |
| octanal | | 0.362 | ± | 0.080 | 0.028 | ± | 0.015 | 0.104 | ± | 0.011 | 0.117 | ± | 0.157 | 0.042 | ± | 0.055 | 0.009 | ± | 0.011 | 0.003 | ± | 0.001 | 0.008 | ± | 0.008 |  |
| 2-methyl-2-octanal | | 0.477 | ± | 0.069 | 0.021 | ± | 0.021 | 0.002 | ± | 0.001 | 0.766 | ± | 0.010 | 0.047 | ± | 0.015 | 0.267 | ± | 0.083 | 0.079 | ± | 0.081 | 0.009 | ± | 0.010 |  |
| nonanal | | 1.515 | ± | 0.515 | 0.018 | ± | 0.005 | 0.751 | ± | 0.261 | 1.644 | ± | 0.457 | 0.091 | ± | 0.121 | 0.011 | ± | 0.006 | 0.013 | ± | 0.004 | 0.143 | ± | 0.020 |  |
| furfural | | 91.014 | ± | 17.720 | 90.124 | ± | 13.702 | 97.692 | ± | 17.260 | 101.720 | ± | 0.611 | 26.133 | ± | 0.773 | 36.823 | ± | 3.768 | 51.550 | ± | 4.172 | 43.482 | ± | 11.129 |  |
| benzaldehyde | | 90.499 | ± | 18.250 | 75.997 | ± | 11.340 | 94.198 | ± | 12.843 | 100.453 | ± | 3.509 | 15.289 | ± | 1.129 | 21.859 | ± | 1.246 | 20.195 | ± | 1.784 | 33.364 | ± | 10.877 |  |
| safranal | | 1.735 | ± | 0.190 | 1.398 | ± | 0.279 | 1.403 | ± | 0.063 | 1.807 | ± | 0.047 | 0.837 | ± | 0.071 | 1.292 | ± | 0.003 | 0.972 | ± | 0.187 | 0.789 | ± | 0.162 |  |
| p-tolualdehyde | | 0.841 | ± | 0.162 | 0.017 | ± | 0.017 | 0.002 | ± | 0.003 | 0.661 | ± | 0.000 | 0.308 | ± | 0.031 | 0.005 | ± | 0.004 | 0.042 | ± | 0.012 | 0.003 | ± | 0.002 |  |
| α,4-dimethyl-3-cyclohexene-1-acetaldehyde | | 1.786 | ± | 0.487 | 0.899 | ± | 0.009 | 1.406 | ± | 0.408 | 0.652 | ± | 0.043 | 0.713 | ± | 0.247 | 1.386 | ± | 0.110 | 1.422 | ± | 0.584 | 0.416 | ± | 0.041 |  |
| *Total* | | 189.163 | ± | 37.324 | 168.546 | ± | 25.300 | 195.820 | ± | 30.771 | 208.467 | ± | 3.620 | 43.480 | ± | 0.197 | 61.676 | ± | 4.987 | 74.292 | ± | 5.499 | 78.243 | ± | 21.887 |  |
|  |  | | | | | | | | | | | | | | | | | | | | | | | | | |
| ***Terpenes, terpenic derivatives and norisoprenoids*** | | | | | | | | | | | | | | | | | | | | | | | | | | |
| unidentified terpene | | 0.166 | ± | 0.118 | 0.943 | ± | 0.358 | 0.372 | ± | 0.063 | 0.243 | ± | 0.265 | 0.215 | ± | 0.044 | 0.217 | ± | 0.187 | 0.285 | ± | 0.022 | 0.160 | ± | 0.016 |  |
| limonene | | 2.789 | ± | 0.030 | 7.857 | ± | 1.153 | 2.988 | ± | 1.150 | 2.331 | ± | 0.005 | 1.756 | ± | 0.734 | 4.032 | ± | 0.759 | 1.892 | ± | 0.231 | 3.054 | ± | 0.134 |  |
| m-cymene | | 0.675 | ± | 0.560 | 1.664 | ± | 0.308 | 0.676 | ± | 0.269 | 0.847 | ± | 0.375 | 0.480 | ± | 0.032 | 0.754 | ± | 0.177 | 0.586 | ± | 0.120 | 1.091 | ± | 0.749 |  |
| prenol | | 0.015 | ± | 0.015 | 0.965 | ± | 0.339 | 0.305 | ± | 0.215 | 0.042 | ± | 0.037 | 0.712 | ± | 0.255 | 2.364 | ± | 0.842 | 1.094 | ± | 0.293 | 1.816 | ± | 0.074 |  |
| α-ionene | | 2.130 | ± | 0.942 | 5.433 | ± | 0.981 | 3.356 | ± | 1.741 | 2.664 | ± | 0.086 | 2.710 | ± | 0.198 | 4.201 | ± | 0.987 | 3.830 | ± | 0.962 | 3.790 | ± | 0.490 |  |
| trans-linalool dioxide | | 0.790 | ± | 0.013 | 1.830 | ± | 0.275 | 0.666 | ± | 0.155 | 0.790 | ± | 0.376 | 0.725 | ± | 0.184 | 1.217 | ± | 0.073 | 0.827 | ± | 0.141 | 0.655 | ± | 0.922 |  |
| β-linalool | | 13.742 | ± | 3.515 | 39.630 | ± | 2.624 | 21.464 | ± | 2.627 | 15.670 | ± | 0.326 | 15.305 | ± | 0.181 | 28.975 | ± | 0.424 | 19.964 | ± | 2.326 | 19.593 | ± | 1.604 |  |
| p-menthen-8-ol | | 3.749 | ± | 1.066 | 10.101 | ± | 0.669 | 5.436 | ± | 1.601 | 4.413 | ± | 0.130 | 3.867 | ± | 0.100 | 7.823 | ± | 0.394 | 5.297 | ± | 0.586 | 4.959 | ± | 0.953 |  |
| trans-geraniol | | 0.291 | ± | 0.065 | 4.174 | ± | 0.365 | 0.781 | ± | 0.156 | 0.323 | ± | 0.082 | 2.547 | ± | 0.033 | 8.922 | ± | 0.406 | 4.593 | ± | 0.416 | 4.586 | ± | 0.507 |  |
| p-mentha-1(7), 8(10)-dien-9-ol | | 0.032 | ± | 0.030 | 1.615 | ± | 0.082 | 0.178 | ± | 0.012 | 0.096 | ± | 0.022 | 0.932 | ± | 0.005 | 2.048 | ± | 0.128 | 1.410 | ± | 0.107 | 1.114 | ± | 0.013 |  |
| eugenol | | 2.425 | ± | 0.516 | 26.830 | ± | 0.111 | 3.910 | ± | 0.643 | 3.200 | ± | 0.124 | 11.051 | ± | 0.397 | 22.447 | ± | 0.111 | 14.671 | ± | 1.352 | 12.971 | ± | 1.513 |  |
| *Total* | | 26.804 | ± | 6.754 | 101.042 | ± | 6.535 | 40.131 | ± | 8.180 | 30.617 | ± | 0.168 | 40.300 | ± | 1.304 | 83.000 | ± | 1.734 | 54.449 | ± | 3.930 | 53.791 | ± | 5.847 |  |
|  |  | | | | | | | | | | | | | | | | | | | | | | | | | |
| ***Acids*** | | | | | | | | | | | | | | | | | | | | | | | | | | |
| acetic acid | | 0.087 | ± | 0.030 | 0.017 | ± | 0.017 | 0.014 | ± | 0.002 | 0.013 | ± | 0.011 | 54.831 | ± | 18.642 | 125.405 | ± | 10.324 | 184.836 | ± | 21.933 | 69.201 | ± | 9.371 |  |
| pivalic acid | | 0.721 | ± | 0.102 | 0.055 | ± | 0.031 | 0.009 | ± | 0.010 | 0.678 | ± | 0.091 | 0.012 | ± | 0.014 | 0.012 | ± | 0.001 | 0.012 | ± | 0.005 | 0.011 | ± | 0.004 |  |
| 4-hydroxybutanoic acid | | 1.029 | ± | 0.192 | 1.304 | ± | 0.072 | 0.943 | ± | 0.195 | 0.982 | ± | 0.187 | 0.743 | ± | 0.037 | 1.422 | ± | 0.017 | 0.901 | ± | 0.154 | 0.370 | ± | 0.141 |  |
| *Total* | | 1.837 | ± | 0.263 | 1.375 | ± | 0.086 | 0.966 | ± | 0.183 | 1.673 | ± | 0.107 | 55.585 | ± | 18.619 | 126.839 | ± | 10.340 | 185.748 | ± | 22.093 | 69.582 | ± | 9.508 |  |
|  |  | | | | | | | | | | | | | | | | | | | | | | | | | |
| ***Esters*** | | | | | | | | | | | | | | | | | | | | | | | | | | |
| propyl acetate | | 0.009 | ± | 0.005 | 1186.731 | ± | 460.827 | 201.607 | ± | 26.688 | 0.008 | ± | 0.005 | 24.680 | ± | 0.422 | 27.876 | ± | 5.804 | 83.370 | ± | 6.871 | 18.539 | ± | 0.273 |  |
| methyl salicylate | | 0.841 | ± | 0.175 | 1.928 | ± | 0.008 | 0.676 | ± | 0.143 | 0.958 | ± | 0.335 | 0.600 | ± | 0.155 | 0.641 | ± | 0.351 | 0.204 | ± | 0.199 | 0.164 | ± | 0.140 |  |
| *Total* | | 0.850 | ± | 0.170 | 1188.660 | ± | 460.835 | 202.284 | ± | 26.544 | 0.966 | ± | 0.340 | 25.279 | ± | 0.266 | 28.517 | ± | 6.155 | 83.574 | ± | 6.673 | 18.703 | ± | 0.133 |  |
|  |  | | | | | | | | | | | | | | | | | | | | | | | | | |
| ***Derivatives of benzene*** | | | | | | | | | | | | | | | | | | | | | | | | | | |
| ethyl benzene | | 0.143 | ± | 0.006 | 0.057 | ± | 0.032 | 0.313 | ± | 0.013 | 0.074 | ± | 0.099 | 0.112 | ± | 0.057 | 0.220 | ± | 0.138 | 0.197 | ± | 0.084 | 0.357 | ± | 0.087 |  |
| styrene | | 0.125 | ± | 0.076 | 0.397 | ± | 0.026 | 0.497 | ± | 0.118 | 0.256 | ± | 0.094 | 0.066 | ± | 0.009 | 0.244 | ± | 0.159 | 0.237 | ± | 0.122 | 1.467 | ± | 1.278 |  |
| m-di-tert-butylbenzene | | 9.004 | ± | 4.001 | 25.514 | ± | 5.636 | 12.572 | ± | 2.402 | 11.422 | ± | 0.409 | 8.313 | ± | 0.294 | 16.358 | ± | 3.493 | 12.119 | ± | 1.264 | 9.563 | ± | 1.992 |  |
| naphthalene | | 0.115 | ± | 0.043 | 1.845 | ± | 0.336 | 0.659 | ± | 0.374 | 0.211 | ± | 0.039 | 1.791 | ± | 0.314 | 3.836 | ± | 0.787 | 8.589 | ± | 0.605 | 0.593 | ± | 0.331 |  |
| 1-1-6-trimethyl-1,2-dihydronaphthalene (TDN) | | 1.683 | ± | 0.588 | 7.825 | ± | 0.359 | 3.402 | ± | 1.785 | 1.910 | ± | 0.220 | 1.506 | ± | 0.102 | 3.981 | ± | 0.969 | 0.671 | ± | 0.090 | 3.046 | ± | 1.050 |  |
| *Total* | | 11.070 | ± | 4.562 | 35.638 | ± | 6.326 | 17.442 | ± | 3.944 | 13.872 | ± | 0.861 | 11.788 | ± | 0.441 | 24.639 | ± | 3.972 | 21.813 | ± | 1.985 | 15.025 | ± | 1.519 |  |
|  |  | | | | | | | | | | | | | | | | | | | | | | | | | |
| ***Others*** | | | | | | | | | | | | | | | | | | | | | | | | | | |
| 2-ethoxy-2-methyl-propane | | 2.377 | ± | 0.073 | 0.248 | ± | 0.266 | 0.008 | ± | 0.003 | 2.582 | ± | 0.253 | 0.393 | ± | 0.028 | 0.568 | ± | 0.133 | 0.631 | ± | 0.061 | 0.024 | ± | 0.009 |  |
| 2-pentylfuran | | 1.580 | ± | 0.616 | 3.929 | ± | 1.013 | 1.307 | ± | 0.606 | 1.597 | ± | 0.509 | 1.230 | ± | 0.283 | 2.298 | ± | 0.187 | 1.676 | ± | 0.158 | 1.219 | ± | 0.043 |  |
| dimethyl trisulfide | | 0.704 | ± | 0.140 | 1.270 | ± | 0.274 | 0.526 | ± | 0.210 | 0.697 | ± | 0.168 | 0.013 | ± | 0.009 | 0.003 | ± | 0.002 | 0.017 | ± | 0.000 | 0.012 | ± | 0.000 |  |
| 4-methyldihydro-2(3H)-furanone | | 0.701 | ± | 0.152 | 0.674 | ± | 0.137 | 1.551 | ± | 0.406 | 1.918 | ± | 0.154 | 0.917 | ± | 0.057 | 1.161 | ± | 0.011 | 0.463 | ± | 0.108 | 0.311 | ± | 0.200 |  |
| 4-ethyl-phenol | | 0.341 | ± | 0.016 | 29.025 | ± | 12.443 | 1.828 | ± | 0.232 | 0.503 | ± | 0.144 | 23.832 | ± | 4.294 | 150.460 | ± | 3.007 | 8.997 | ± | 3.734 | 106.967 | ± | 6.068 |  |
| *Total* | | 5.703 | ± | 0.686 | 35.146 | ± | 11.029 | 5.219 | ± | 1.032 | 7.297 | ± | 0.892 | 26.384 | ± | 4.614 | 154.491 | ± | 2.675 | 11.784 | ± | 3.845 | 108.532 | ± | 5.902 |  |
|  | |  |  |  |  |  |  |  |  |  |  |  |  |  |  |  |  |  |  |  |  |  |  |  |  |  |
| ***Total*** | | 319.136 | ± | 68.866 | 2060.672 | ± | 535.185 | 579.758 | ± | 36.858 | 365.044 | ± | 3.750 | 387.242 | ± | 25.660 | 801.971 | ± | 34.964 | 878.517 | ± | 80.494 | 530.291 | ± | 43.821 |  |

Data are the mean values of three independent experiments ± standard deviation.

Table S3B. **Volatile compounds.** Concentration (ng/mL) of volatile compounds identified in sweet cherry juices fermented with L. plantarum (POM1, C1, 1LE1, 285), L. rhamnosus (2360). L. paracasei (4186) and in controls (juices treated at 30°C and 37°C) after storage (14 days).

| Compound | | 37 °C | | | 2360 | | | 4186 | | | 30 °C | | | 1LE1 | | | 285 | | | C1 | | | POM1 | | |
| --- | --- | --- | --- | --- | --- | --- | --- | --- | --- | --- | --- | --- | --- | --- | --- | --- | --- | --- | --- | --- | --- | --- | --- | --- | --- |
|  |  | | | | | | | | | | | | | | | | | | | | | | | | |
| ***Ketones*** | | | | | | | | | | | | | | | | | | | | | | | | | |
| acetone | | 17.019 | ± | 0.334 | 7.165 | ± | 0.532 | 18.316 | ± | 3.144 | 11.018 | ± | 0.266 | 23.464 | ± | 0.394 | 18.394 | ± | 2.564 | 10.602 | ± | 0.392 | 4.140 | ± | 0.988 |
| 2-heptanone | | 0.412 | ± | 0.166 | 1.191 | ± | 0.484 | 0.744 | ± | 0.077 | 0.703 | ± | 0.240 | 0.491 | ± | 0.301 | 0.453 | ± | 0.276 | 1.662 | ± | 0.675 | 0.011 | ± | 0.003 |
| 4-methyl-2-heptanone | | 4.280 | ± | 1.064 | 0.008 | ± | 0.009 | 0.023 | ± | 0.005 | 1.116 | ± | 1.570 | 1.962 | ± | 0.107 | 0.990 | ± | 0.426 | 1.740 | ± | 0.004 | 0.010 | ± | 0.005 |
| ethyl isoamyl ketone | | 0.005 | ± | 0.002 | 0.508 | ± | 0.007 | 1.256 | ± | 0.225 | 0.002 | ± | 0.002 | 0.001 | ± | 0.001 | 0.004 | ± | 0.001 | 0.168 | ± | 0.075 | 0.006 | ± | 0.001 |
| 1-decen-3-one | | 0.352 | ± | 0.175 | 0.003 | ± | 0.004 | 0.214 | ± | 0.037 | 0.330 | ± | 0.147 | 0.417 | ± | 0.179 | 0.057 | ± | 0.051 | 0.152 | ± | 0.144 | 0.004 | ± | 0.004 |
| acetoin | | 0.004 | ± | 0.005 | 180.078 | ± | 22.938 | 65.013 | ± | 1.293 | 0.004 | ± | 0.000 | 580.569 | ± | 61.646 | 161.905 | ± | 50.669 | 1058.883 | ± | 222.855 | 63.563 | ± | 22.691 |
| 2-nonanone | | 0.003 | ± | 0.004 | 2.868 | ± | 1.433 | 1.264 | ± | 0.915 | 0.010 | ± | 0.010 | 0.137 | ± | 0.007 | 0.048 | ± | 0.000 | 0.079 | ± | 0.053 | 0.019 | ± | 0.005 |
| *Total* | | 22.075 | ± | 1.396 | 191.821 | ± | 21.541 | 86.831 | ± | 2.956 | 13.184 | ± | 1.940 | 607.041 | ± | 61.244 | 181.852 | ± | 53.434 | 1073.285 | ± | 223.942 | 67.753 | ± | 23.683 |
|  |  | | | | | | | | | | | | | | | | | | | | | | | | |
| ***Alcohols*** | | | | | | | | | | | | | | | | | | | | | | | | | |
| ethanol | | 10.279 | ± | 0.187 | 3.171 | ± | 0.818 | 13.702 | ± | 2.820 | 6.607 | ± | 0.829 | 17.334 | ± | 0.324 | 12.610 | ± | 1.060 | 12.145 | ± | 1.982 | 4.724 | ± | 1.259 |
| isopentyl alcohol | | 1.236 | ± | 0.515 | 0.293 | ± | 0.400 | 1.380 | ± | 0.162 | 0.006 | ± | 0.004 | 3.879 | ± | 0.132 | 1.658 | ± | 0.743 | 2.324 | ± | 0.223 | 0.709 | ± | 0.078 |
| 3-pentenol | | 0.009 | ± | 0.004 | 0.004 | ± | 0.000 | 0.022 | ± | 0.018 | 1.104 | ± | 0.268 | 0.013 | ± | 0.010 | 0.029 | ± | 0.019 | 0.010 | ± | 0.001 | 0.005 | ± | 0.001 |
| isobutenylcarbinol | | 2.038 | ± | 0.380 | 2.111 | ± | 1.091 | 6.378 | ± | 0.091 | 0.009 | ± | 0.008 | 11.220 | ± | 0.895 | 5.171 | ± | 1.246 | 3.794 | ± | 0.915 | 2.769 | ± | 0.097 |
| hexanol | | 1.980 | ± | 0.143 | 1.048 | ± | 0.185 | 4.093 | ± | 0.652 | 1.488 | ± | 0.016 | 13.582 | ± | 1.441 | 8.096 | ± | 1.266 | 7.732 | ± | 0.582 | 2.438 | ± | 0.691 |
| (E)-2-hexen-1-ol | | 30.927 | ± | 0.265 | 12.219 | ± | 1.310 | 43.517 | ± | 9.104 | 20.374 | ± | 0.288 | 73.581 | ± | 10.961 | 49.312 | ± | 4.692 | 44.018 | ± | 5.733 | 19.185 | ± | 6.566 |
| amyl vinyl carbinol | | 1.074 | ± | 0.709 | 0.365 | ± | 0.020 | 1.198 | ± | 0.555 | 1.108 | ± | 0.618 | 2.037 | ± | 0.360 | 1.226 | ± | 0.190 | 1.224 | ± | 0.292 | 0.300 | ± | 0.016 |
| 1-heptanol | | 0.036 | ± | 0.008 | 0.074 | ± | 0.028 | 0.350 | ± | 0.023 | 0.070 | ± | 0.039 | 5.259 | ± | 0.861 | 1.880 | ± | 0.038 | 2.282 | ± | 0.612 | 1.145 | ± | 0.357 |
| 2-ethyl-1-hexanol | | 5.300 | ± | 0.280 | 3.843 | ± | 0.840 | 8.464 | ± | 0.821 | 3.854 | ± | 0.373 | 21.104 | ± | 7.591 | 11.307 | ± | 3.796 | 7.173 | ± | 0.514 | 3.125 | ± | 2.427 |
| octanol | | 9.593 | ± | 0.892 | 3.233 | ± | 0.490 | 18.840 | ± | 4.930 | 6.970 | ± | 0.015 | 27.485 | ± | 5.391 | 14.897 | ± | 1.322 | 21.430 | ± | 2.702 | 5.780 | ± | 2.838 |
| 1-nonanol | | 0.087 | ± | 0.076 | 0.215 | ± | 0.129 | 12.439 | ± | 5.094 | 0.342 | ± | 0.254 | 22.386 | ± | 5.568 | 8.428 | ± | 0.357 | 16.863 | ± | 4.236 | 2.360 | ± | 1.378 |
| 2-furanmethanol | | 1.575 | ± | 0.281 | 6.113 | ± | 1.576 | 7.858 | ± | 0.346 | 0.613 | ± | 0.026 | 14.657 | ± | 4.815 | 10.287 | ± | 1.807 | 27.780 | ± | 2.299 | 4.149 | ± | 1.524 |
| decanol | | 5.322 | ± | 0.595 | 2.025 | ± | 0.451 | 16.246 | ± | 3.482 | 3.158 | ± | 0.348 | 25.055 | ± | 3.666 | 13.155 | ± | 1.883 | 23.224 | ± | 4.829 | 5.609 | ± | 3.053 |
| benzene methanol | | 146.075 | ± | 6.008 | 79.259 | ± | 21.526 | 250.467 | ± | 32.932 | 99.225 | ± | 1.022 | 462.867 | ± | 76.120 | 311.721 | ± | 47.740 | 327.123 | ± | 54.469 | 116.204 | ± | 42.234 |
| benzene ethanol | | 7.855 | ± | 1.017 | 4.045 | ± | 0.824 | 13.254 | ± | 0.767 | 5.818 | ± | 0.339 | 31.874 | ± | 4.004 | 21.152 | ± | 3.347 | 23.079 | ± | 4.527 | 7.650 | ± | 2.463 |
| *Total* | | 223.386 | ± | 5.742 | 118.019 | ± | 25.871 | 398.208 | ± | 60.553 | 150.746 | ± | 0.615 | 732.332 | ± | 120.329 | 470.929 | ± | 60.746 | 520.201 | ± | 81.637 | 176.153 | ± | 64.981 |
|  |  | | | | | | | | | | | | | | | | | | | | | | | | |
| ***Aldehydes*** | | | | | | | | | | | | | | | | | | | | | | | | | |
| isovaleraldehyde | | 4.504 | ± | 0.127 | 0.008 | ± | 0.008 | 1.422 | ± | 0.492 | 0.995 | ± | 0.130 | 0.060 | ± | 0.033 | 0.046 | ± | 0.020 | 0.141 | ± | 0.112 | 0.020 | ± | 0.008 |
| octanal | | 1.880 | ± | 0.964 | 0.011 | ± | 0.015 | 0.009 | ± | 0.009 | 2.020 | ± | 0.905 | 0.017 | ± | 0.019 | 0.017 | ± | 0.003 | 0.007 | ± | 0.000 | 0.011 | ± | 0.003 |
| 2-methyl-2-octanal | | 2.054 | ± | 0.898 | 0.033 | ± | 0.015 | 0.067 | ± | 0.059 | 0.889 | ± | 0.376 | 0.586 | ± | 0.438 | 0.171 | ± | 0.182 | 0.223 | ± | 0.240 | 0.019 | ± | 0.007 |
| nonanal | | 7.013 | ± | 1.434 | 0.160 | ± | 0.212 | 0.590 | ± | 0.824 | 9.584 | ± | 0.786 | 0.070 | ± | 0.034 | 0.785 | ± | 0.988 | 0.677 | ± | 0.919 | 0.017 | ± | 0.008 |
| furfural | | 265.690 | ± | 8.002 | 8.274 | ± | 2.030 | 290.648 | ± | 57.628 | 170.423 | ± | 3.198 | 78.144 | ± | 3.941 | 81.231 | ± | 36.107 | 139.988 | ± | 0.807 | 13.842 | ± | 6.288 |
| benzaldehyde | | 267.490 | ± | 2.961 | 14.710 | ± | 3.707 | 279.968 | ± | 63.993 | 175.687 | ± | 5.154 | 81.874 | ± | 4.331 | 93.758 | ± | 34.784 | 80.357 | ± | 2.603 | 18.478 | ± | 7.338 |
| safranal | | 4.789 | ± | 0.189 | 0.791 | ± | 0.197 | 3.700 | ± | 1.130 | 3.176 | ± | 0.025 | 4.299 | ± | 0.723 | 3.013 | ± | 0.605 | 2.833 | ± | 0.406 | 0.966 | ± | 0.440 |
| p-tolualdehyde | | 3.402 | ± | 0.490 | 0.010 | ± | 0.006 | 0.093 | ± | 0.088 | 1.988 | ± | 0.668 | 0.588 | ± | 0.073 | 0.817 | ± | 0.582 | 0.018 | ± | 0.002 | 0.013 | ± | 0.004 |
| α.4-dimethyl-3-cyclohexene-1-acetaldehyde | | 2.471 | ± | 0.377 | 0.661 | ± | 0.433 | 2.069 | ± | 0.054 | 1.496 | ± | 0.215 | 4.986 | ± | 1.873 | 2.650 | ± | 0.779 | 3.060 | ± | 0.577 | 1.386 | ± | 0.732 |
| *Total* | | 559.293 | ± | 8.135 | 24.659 | ± | 5.260 | 578.568 | ± | 122.520 | 366.258 | ± | 6.051 | 170.624 | ± | 2.736 | 182.487 | ± | 72.034 | 227.304 | ± | 1.990 | 34.753 | ± | 14.790 |
|  |  | | | | | | | | | | | | | | | | | | | | | | | | |
| ***Terpenes. terpenic derivatives and norisoprenoids*** | | | | | | | | | | | | | | | | | | | | | | | | | |
| unidentified terpene | | 1.083 | ± | 0.488 | 0.024 | ± | 0.004 | 0.670 | ± | 0.212 | 0.197 | ± | 0.046 | 1.026 | ± | 0.361 | 0.386 | ± | 0.024 | 0.521 | ± | 0.283 | 0.310 | ± | 0.059 |
| limonene | | 5.535 | ± | 1.386 | 2.747 | ± | 0.054 | 9.462 | ± | 2.056 | 3.048 | ± | 0.906 | 10.710 | ± | 1.511 | 7.443 | ± | 1.183 | 8.309 | ± | 1.944 | 2.989 | ± | 0.383 |
| m-cymene | | 1.128 | ± | 0.266 | 0.307 | ± | 0.417 | 2.064 | ± | 0.182 | 0.830 | ± | 0.021 | 2.410 | ± | 0.372 | 4.658 | ± | 3.293 | 2.198 | ± | 0.325 | 0.704 | ± | 0.316 |
| prenol | | 0.044 | ± | 0.037 | 0.442 | ± | 0.091 | 0.699 | ± | 0.426 | 0.019 | ± | 0.003 | 3.679 | ± | 0.221 | 6.079 | ± | 0.513 | 2.406 | ± | 0.434 | 1.643 | ± | 0.235 |
| α-ionene | | 4.719 | ± | 0.343 | 2.858 | ± | 0.133 | 9.821 | ± | 0.952 | 3.848 | ± | 0.951 | 14.112 | ± | 4.440 | 8.683 | ± | 1.158 | 11.341 | ± | 0.388 | 3.355 | ± | 1.964 |
| trans-linalool dioxide | | 1.645 | ± | 0.056 | 0.845 | ± | 0.013 | 2.530 | ± | 0.650 | 0.793 | ± | 1.114 | 4.340 | ± | 0.907 | 1.897 | ± | 0.251 | 2.552 | ± | 0.367 | 0.924 | ± | 0.292 |
| β-linalool | | 41.464 | ± | 3.599 | 19.467 | ± | 2.875 | 70.918 | ± | 15.120 | 27.205 | ± | 2.210 | 92.675 | ± | 20.477 | 57.517 | ± | 9.385 | 68.307 | ± | 4.195 | 22.431 | ± | 8.860 |
| p-menthen-8-ol | | 11.039 | ± | 0.950 | 4.659 | ± | 1.011 | 18.832 | ± | 3.255 | 6.965 | ± | 0.034 | 25.450 | ± | 4.907 | 15.603 | ± | 1.759 | 19.160 | ± | 1.918 | 5.929 | ± | 2.607 |
| trans-geraniol | | 1.472 | ± | 0.039 | 1.798 | ± | 0.348 | 3.871 | ± | 0.457 | 1.012 | ± | 0.054 | 17.614 | ± | 2.150 | 16.240 | ± | 0.618 | 16.176 | ± | 2.694 | 7.563 | ± | 3.181 |
| p-mentha-1(7). 8(10)-dien-9-ol | | 0.143 | ± | 0.169 | 0.981 | ± | 0.071 | 1.057 | ± | 0.125 | 0.094 | ± | 0.070 | 5.324 | ± | 1.211 | 2.963 | ± | 0.206 | 4.736 | ± | 0.836 | 1.484 | ± | 0.798 |
| eugenol | | 6.731 | ± | 0.296 | 11.209 | ± | 2.994 | 19.468 | ± | 0.240 | 4.680 | ± | 0.167 | 64.981 | ± | 10.397 | 36.587 | ± | 3.605 | 52.905 | ± | 11.791 | 17.755 | ± | 5.498 |
| *Total* | | 75.003 | ± | 4.020 | 45.338 | ± | 7.737 | 139.391 | ± | 17.919 | 48.690 | ± | 3.125 | 242.321 | ± | 46.952 | 158.056 | ± | 13.972 | 188.611 | ± | 20.721 | 65.086 | ± | 24.193 |
|  |  | | | | | | | | | | | | | | | | | | | | | | | | |
| ***Acids*** | | | | | | | | | | | | | | | | | | | | | | | | | |
| acetic acid | | 0.024 | ± | 0.009 | 228.633 | ± | 71.471 | 0.354 | ± | 0.192 | 0.013 | ± | 0.009 | 535.465 | ± | 92.920 | 305.391 | ± | 5.248 | 737.615 | ± | 193.995 | 257.568 | ± | 75.381 |
| pivalic acid | | 0.009 | ± | 0.002 | 0.022 | ± | 0.019 | 0.042 | ± | 0.030 | 0.010 | ± | 0.002 | 0.058 | ± | 0.017 | 0.019 | ± | 0.006 | 0.232 | ± | 0.080 | 0.065 | ± | 0.066 |
| 4-hydroxybutanoic acid | | 1.925 | ± | 0.150 | 0.745 | ± | 0.368 | 2.162 | ± | 0.084 | 1.422 | ± | 0.098 | 3.665 | ± | 0.433 | 2.802 | ± | 0.191 | 2.784 | ± | 0.428 | 0.579 | ± | 0.084 |
| *Total* | | 1.957 | ± | 0.143 | 229.400 | ± | 71.820 | 2.557 | ± | 0.138 | 1.445 | ± | 0.108 | 539.188 | ± | 93.370 | 308.211 | ± | 5.064 | 740.631 | ± | 194.502 | 258.211 | ± | 75.531 |
|  |  | | | | | | | | | | | | | | | | | | | | | | | | |
| ***Esters*** | | | | | | | | | | | | | | | | | | | | | | | | | |
| propyl acetate | | 0.026 | ± | 0.004 | 509.194 | ± | 12.345 | 1230.067 | ± | 222.171 | 0.015 | ± | 0.014 | 160.882 | ± | 2.895 | 61.035 | ± | 14.937 | 281.546 | ± | 28.411 | 16.706 | ± | 5.548 |
| methyl salicylate | | 1.221 | ± | 0.585 | 0.821 | ± | 0.678 | 1.261 | ± | 0.432 | 1.569 | ± | 0.166 | 3.305 | ± | 0.101 | 1.598 | ± | 1.776 | 1.668 | ± | 0.212 | 0.492 | ± | 0.025 |
| *Total* | | 1.247 | ± | 0.581 | 510.015 | ± | 13.023 | 1231.328 | ± | 222.604 | 1.585 | ± | 0.180 | 164.187 | ± | 2.996 | 62.634 | ± | 16.714 | 283.214 | ± | 28.623 | 17.199 | ± | 5.573 |
|  |  | | | | | | | | | | | | | | | | | | | | | | | | |
| ***Derivatives of benzene*** | | | | | | | | | | | | | | | | | | | | | | | | | |
| ethyl benzene | | 0.313 | ± | 0.131 | 0.276 | ± | 0.108 | 0.259 | ± | 0.202 | 0.066 | ± | 0.030 | 0.935 | ± | 0.534 | 0.979 | ± | 0.598 | 0.255 | ± | 0.195 | 0.123 | ± | 0.088 |
| styrene | | 0.574 | ± | 0.092 | 0.792 | ± | 0.118 | 0.842 | ± | 0.147 | 0.257 | ± | 0.275 | 2.261 | ± | 2.241 | 3.107 | ± | 3.482 | 0.575 | ± | 0.138 | 0.161 | ± | 0.022 |
| m-di-tert-butylbenzene | | 22.624 | ± | 1.371 | 12.075 | ± | 1.783 | 41.505 | ± | 7.869 | 12.545 | ± | 0.661 | 53.579 | ± | 13.912 | 32.527 | ± | 8.208 | 49.598 | ± | 7.273 | 13.282 | ± | 6.780 |
| naphthalene | | 0.463 | ± | 0.003 | 0.471 | ± | 0.281 | 0.730 | ± | 0.098 | 0.314 | ± | 0.125 | 1.782 | ± | 0.318 | 0.728 | ± | 0.141 | 31.024 | ± | 7.704 | 2.946 | ± | 1.377 |
| 1-1-6-trimethyl-1.2-dihydronaphthalene (TDN) | | 5.129 | ± | 0.668 | 3.157 | ± | 0.772 | 13.343 | ± | 3.346 | 3.228 | ± | 0.637 | 15.379 | ± | 5.184 | 9.093 | ± | 1.748 | 3.737 | ± | 1.724 | 2.750 | ± | 1.848 |
| *Total* | | 29.103 | ± | 2.258 | 16.772 | ± | 2.846 | 56.679 | ± | 11.466 | 16.409 | ± | 0.406 | 73.936 | ± | 21.555 | 46.434 | ± | 14.178 | 85.190 | ± | 13.196 | 19.263 | ± | 9.939 |
|  |  | | | | | | | | | | | | | | | | | | | | | | | | |
| ***Others*** | | | | | | | | | | | | | | | | | | | | | | | | | |
| 2-ethoxy-2-methyl-propane | | 7.172 | ± | 0.012 | 0.030 | ± | 0.001 | 0.025 | ± | 0.010 | 4.289 | ± | 0.099 | 4.147 | ± | 0.641 | 2.023 | ± | 0.159 | 1.993 | ± | 1.045 | 0.023 | ± | 0.005 |
| 2-pentylfuran | | 2.776 | ± | 1.974 | 0.891 | ± | 0.204 | 6.134 | ± | 0.909 | 1.610 | ± | 0.076 | 6.036 | ± | 1.548 | 8.781 | ± | 1.384 | 4.511 | ± | 1.086 | 1.559 | ± | 0.878 |
| dimethyl trisulfide | | 1.777 | ± | 0.421 | 0.610 | ± | 0.114 | 1.821 | ± | 0.286 | 1.157 | ± | 0.226 | 0.009 | ± | 0.007 | 0.016 | ± | 0.012 | 0.041 | ± | 0.036 | 0.013 | ± | 0.000 |
| 4-methyldihydro-2(3H)-furanone | | 3.750 | ± | 1.128 | 0.516 | ± | 0.710 | 1.582 | ± | 1.008 | 1.688 | ± | 0.497 | 5.612 | ± | 0.747 | 1.646 | ± | 0.683 | 0.950 | ± | 0.945 | 0.287 | ± | 0.104 |
| 4-ethyl-phenol | | 0.848 | ± | 0.141 | 3.210 | ± | 0.751 | 21.854 | ± | 5.943 | 0.608 | ± | 0.144 | 129.556 | ± | 2.648 | 281.248 | ± | 21.113 | 12.287 | ± | 0.017 | 136.453 | ± | 43.612 |
| *Total* | | 16.324 | ± | 1.396 | 5.257 | ± | 1.781 | 31.416 | ± | 3.729 | 9.352 | ± | 0.104 | 145.360 | ± | 1.001 | 293.713 | ± | 20.560 | 19.782 | ± | 1.133 | 138.335 | ± | 44.380 |
|  | |  |  |  |  |  |  |  |  |  |  |  |  |  |  |  |  |  |  |  |  |  |  |  |  |
| ***Total*** | | 928.388 | ± | 19.716 | 1141.280 | ± | 149.879 | 2524.976 | ± | 434.426 | 607.670 | ± | 3.670 | 2674.990 | ± | 348.181 | 1704.317 | ± | 246.572 | 3138.218 | ± | 565.745 | 776.754 | ± | 263.071 |

Data are the mean values of three independent experiments ± standard deviation.
