# Supplementary material for: Use of Dairy and Plant-Derived Lactobacilli as Starters for Cherry Juice Fermentation
Source: Nutrients. 2019 Jan 22;11(2):213. doi: 10.3390/nu11020213 (PMC6412669; doi:10.3390/nu11020213)
Supplement: Supplementary file 1 [file nutrients-11-00213-s001.zip › Supplememntary materials/Supplementary table S4.docx]

| Table S4. **Identification of phenolic compounds.** Chromatographic and mass spectral characteristics of phenolic compounds detected in started and unstarted cherry juices. For quantified compounds a specific variable, used in PCA analyses, was assigned. | | | | | | | |
| --- | --- | --- | --- | --- | --- | --- | --- |
|  | **Compound** | **RT** | **[M-H]^-^**  **(m/z)** | **MS^2^ ion fragments (m/z)** | **MS^3^ ion fragments (m/z)** | **Quantification** | **Standard** |
| P59 | *p*-Hydroxyphenyllactic acid | 4.18 | 181 | **163**, 135 |  | SRM | *p*-Hydroxyphenyllactic acid |
| P60 | Phenyllactic acid | 6.21 | 165 | **147**, 119 |  | SRM | Phenyllactic acid |
| P61 | 3-*O*-caffeoylquinic acid | 4.00 | 353 | **191**, 179, 173 |  | SRM | 3-*O*-caffeoylquinic acid |
| P62 | 5-*O*-caffeoylquinic acid | 4.70 | 353 | **191**, 179. 173 |  | SRM | 5-*O*-caffeoylquinic acid |
| P63 | 4-*O*-caffeoylquinic acid | 4.80 | 353 | **173**, 179, 191 |  | SRM | 4-*O*-caffeoylquinic acid |
| P64 | Caffeoylquinic acid | 5.16 | 353 | **191**, 179, 173 |  | SRM | 5-*O*-caffeoylquinic acid |
| P65 | Caffeic acid | 5.15 | 179 | 135 |  | SIM | Caffeic acid |
| P66 | Dicaffeoylquinic acid (1) | 6.62 | 515 | **353**, 179 |  | SRM | 5-*O*-caffeoylquinic acid |
| P67 | Dicaffeoylquinic acid (2) | 6.60 | 515 | **353** | 191, 179, 135, 173 | SRM | 5-*O*-caffeoylquinic acid |
| P68 | Dicaffeoylquinic acid (3) | 6.84 | 515 | **353**, 179, 191 |  | SRM | 5-*O*-caffeoylquinic acid |
| P69 | Coumaroylquinic acid (1) | 4.58 | 337 | **163**, 191, 173 | 163: 119 | SRM | 3-*O*-caffeoylquinic acid |
| P70 | Coumaroylquinic acid (2) | 5.20 | 337 | **173**, 191, 163 |  | SRM | 4-*O*-caffeoylquinic acid |
| P71 | Coumaroylquinic acid (3) | 5.33 | 337 | **173**, 191, 163 | 173: 93, 111, 71 | SRM | 4-*O*-caffeoylquinic acid |
| P72 | Coumaroylquinic acid (4) | 5.75 | 337 | **191**, 163, 173 | 191: 127, 173, 109, 93, 85, 111 | SRM | 5-*O*-caffeoylquinic acid |
| P73 | *p*-coumaric acid | 6.12 | 163 |  |  | SIM | *p*-coumaric acid |
| P74 | Feruloylquinic acid (1) | 4.87 | 367 | **193**, 173, 191, | 193: 134, 149, 178 | SRM | 3-*O*-caffeoylquinic acid |
| P75 | Coumaroylquinic acid lactone | 6.58 | 319 | **145**, 119, 163 |  | SRM | 3-*O*-caffeoylquinic acid |
| P76 | Coumaric acid-*O*-hexoside | 4.11 | 325 | 163, 119 |  | SIM | *p*-coumaric acid |
| P77 | Caffeic acid-*O*-hexoside | 4.55 | 341 | 179, 135 |  | SIM | Caffeic acid |
| P78 | Caffeoylquinic acid-*O*-hexoside | 3.77 | 515 | 341,179**, 353**, 173, 191 | 341: 179, 135 | SRM | 3-*O*-caffeoylquinic acid |
| P79 | Luteolin | 8.24 | 285 | 241, 243, 175, 199, 217, 151 |  | SIM | Luteolin |
| P80 | Dihdroxybenzoic acid-*O*-hexoside | 3.36 | 315 | 153, 109 | 153: 109 | SIM | Protocatecuic acid |
| P81 | Protocatecuic acid | 3.80 | 153 | 109 |  | SIM | Protocatecuic acid |
| P82 | Quercetin | 8.30 | 301 | 179, 151, 257, 273 |  | SIM | Quercetin dihydrate |
| P83 | Rutin | 5.97 | 609 | **301**, 343 | 301: 179, 151, 257, 273 | SRM | Rutin hydrate |
| P84 | Quercetin-3-*O*-glucoside | 6.23 | 463 | **301**, 179 | 301: 179, 151, 257, 273 | SRM | Rutin hydrate |
| P85 | Kaempferol-*O*-rutinoside | 6.41 | 593 | **285**, 327 | 285: 257, 241, 239, 169, 187, 151 | SRM | Rutin hydrate |
| P86 | (+)-Catechin | 4.71 | 289 | 245, 205, 179, 271, 137 |  | SIM | (+)-Catechin |
| P87 | (-)-Epicatechin | 5.25 | 289 | 245, 205, 179, 271, 137 |  | SIM | (-)-Epicatechin |
| P88 | Dihydrocaffeic acid | 5.29 | 181 | **137**, 109, 119 |  | SRM | Dihydrocaffeic acid |
| NQ | (±)-Naringenin | 9.10 | 271 | 151, 177, 107, 201 |  | SIM | (±)-Naringenin |
| NQ | Kaempferol | 9.32 | 285 | 257, 241, 239, 169, 187, 151 |  | SIM | Kaempferol |
| NQ | Feruloylquinic acid (2) | 5.59 | 367 | **173**, 191, 193 | 173: 93, 111, 71 | SRM | 4-*O*-caffeoylquinic acid |
| NQ | Coumaroylquinic acid-*O*-hexoside | 3.50 | 499 | **325**, 163 | 163: 119 | SRM | 3-*O*-caffeoylquinic acid |

NQ: not quantified. Fragment ions are listed in order of their relative abundances. MS^2^ ions in bold were used as quantifier ions
