# Supplementary material for: Use of Dairy and Plant-Derived Lactobacilli as Starters for Cherry Juice Fermentation
Source: Nutrients. 2019 Jan 22;11(2):213. doi: 10.3390/nu11020213 (PMC6412669; doi:10.3390/nu11020213)
Supplement: Supplementary file 1 [file nutrients-11-00213-s001.zip › Supplememntary materials/Supplementary table S5.docx]

| Table S5. **Phenolic profile.** Concentration (µg/mL) of phenolic compounds detected in unfermented (37°C and 30°C) and fermented with *L. rhamnosus* 2360, *L. paracasei* 4186, *L. plantarum* 1LE1, 285, C1 and POM1 cherry juice after 48 hours of fermentation and further storage (14 days). | | | | | | | | | | | | | | | | | | | | | | | | | | | | | | | |
| --- | --- | --- | --- | --- | --- | --- | --- | --- | --- | --- | --- | --- | --- | --- | --- | --- | --- | --- | --- | --- | --- | --- | --- | --- | --- | --- | --- | --- | --- | --- | --- |
|  | ***48 hours*** | | | | | | | | | | | | | | | | | | | | | | | | | | | | | | |
| **Compounds** | **37°C** | | |  | **2360** | | |  | **4186** | | |  | **30°C** | | |  | **1LE1** | | |  | **285** | | |  | **C1** | | |  | **POM1** | | |
|  |  |  |  |  |  |  |  |  |  |  |  |  |  |  |  |  |  |  |  |  |  |  |  |  |  |  |  |  |  |  |  |
| **Phenyllactic acids** |  |  |  |  |  |  |  |  |  |  |  |  |  |  |  |  |  |  |  |  |  |  |  |  |  |  |  |  |  |  |  |
| *p*-Hydroxyphenyllactic acid | ND | | |  | 0.986 | ± | 0.121 |  | 0.209 | ± | 0.047 |  | ND | | |  | 1.229 | ± | 0.187 |  | 1.462 | ± | 0.089 |  | 1.241 | ± | 0.148 |  | 1.134 | ± | 0.125 |
| Phenyllactic acid | ND | | |  | 0.483 | ± | 0.062 |  | 0.449 | ± | 0.024 |  | ND | | |  | 2.098 | ± | 0.187 |  | 1.905 | ± | 0.150 |  | 1.219 | ± | 0.076 |  | 1.930 | ± | 0.026 |
| **Total** | ND | | |  | 1.469 | ± | 0.156 |  | 0.659 | ± | 0.034 |  | ND | | |  | 3.327 | ± | 0.365 |  | 3.367 | ± | 0.157 |  | 2.461 | ± | 0.166 |  | 3.064 | ± | 0.120 |
|  |  |  |  |  |  |  |  |  |  |  |  |  |  |  |  |  |  |  |  |  |  |  |  |  |  |  |  |  |  |  |  |
| **Hydroxycinnamic acids** |  |  |  |  |  |  |  |  |  |  |  |  |  |  |  |  |  |  |  |  |  |  |  |  |  |  |  |  |  |  |  |
| 3-*O*-Caffeoylquinic acid | 19.407 | ± | 2.234 |  | 17.470 | ± | 1.020 |  | 19.323 | ± | 0.343 |  | 17.706 | ± | 0.563 |  | 20.469 | ± | 1.536 |  | 18.452 | ± | 0.693 |  | 18.305 | ± | 1.324 |  | 17.164 | ± | 0.776 |
| 5-*O*-Caffeoylquinic acid | 2.652 | ± | 0.183 |  | 2.964 | ± | 0.339 |  | 3.660 | ± | 0.233 |  | 2.446 | ± | 0.099 |  | 3.441 | ± | 0.321 |  | 3.361 | ± | 0.062 |  | 2.898 | ± | 0.180 |  | 2.968 | ± | 0.214 |
| 4-*O*-Caffeoylquinic acid | 8.418 | ± | 1.077 |  | 8.347 | ± | 0.295 |  | 10.304 | ± | 0.248 |  | 7.101 | ± | 0.001 |  | 9.778 | ± | 1.261 |  | 8.887 | ± | 0.580 |  | 9.028 | ± | 0.176 |  | 8.683 | ± | 0.817 |
| Caffeoylquinic acid | 0.036 | ± | 0.002 |  | 0.040 | ± | 0.004 |  | 0.041 | ± | 0.003 |  | 0.039 | ± | 0.003 |  | 0.044 | ± | 0.009 |  | 0.044 | ± | 0.007 |  | 0.038 | ± | 0.003 |  | 0.039 | ± | 0.003 |
| Caffeic acid | 0.633 | ± | 0.025 |  | 0.743 | ± | 0.038 |  | 0.722 | ± | 0.030 |  | 0.586 | ± | 0.033 |  | 0.406 | ± | 0.270 |  | ND | | |  | 0.305 | ± | 0.040 |  | 0.062 | ± | 0.014 |
| Dicaffeoylquinic acid (1) | 0.004 | ± | 0.004 |  | 0.008 | ± | 0.001 |  | 0.011 | ± | 0.005 |  | 0.004 | ± | 0.000 |  | 0.009 | ± | 0.003 |  | 0.008 | ± | 0.003 |  | 0.008 | ± | 0.002 |  | 0.010 | ± | 0.002 |
| Dicaffeoylquinic acid (2) | 0.010 | ± | 0.004 |  | 0.025 | ± | 0.002 |  | 0.028 | ± | 0.006 |  | 0.011 | ± | 0.001 |  | 0.035 | ± | 0.002 |  | 0.032 | ± | 0.007 |  | 0.030 | ± | 0.000 |  | 0.023 | ± | 0.005 |
| Dicaffeoylquinic acid (3) | 0.025 | ± | 0.005 |  | 0.037 | ± | 0.006 |  | 0.043 | ± | 0.014 |  | 0.020 | ± | 0.003 |  | 0.049 | ± | 0.010 |  | 0.037 | ± | 0.001 |  | 0.039 | ± | 0.006 |  | 0.036 | ± | 0.002 |
| Coumaroylquinic acid (1) | 21.714 | ± | 2.529 |  | 24.020 | ± | 1.087 |  | 24.680 | ± | 1.949 |  | 21.828 | ± | 0.354 |  | 24.564 | ± | 2.152 |  | 24.898 | ± | 1.265 |  | 22.903 | ± | 0.415 |  | 23.357 | ± | 0.853 |
| Coumaroylquinic acid (2) | 6.537 | ± | 0.266 |  | 6.466 | ± | 0.481 |  | 7.647 | ± | 0.320 |  | 5.547 | ± | 0.502 |  | 7.478 | ± | 0.865 |  | 6.398 | ± | 0.473 |  | 6.245 | ± | 0.104 |  | 6.563 | ± | 0.106 |
| Coumaroylquinic acid (3) | 3.746 | ± | 0.151 |  | 4.002 | ± | 0.597 |  | 4.584 | ± | 0.116 |  | 3.445 | ± | 0.308 |  | 4.220 | ± | 0.194 |  | 4.053 | ± | 0.250 |  | 3.811 | ± | 0.132 |  | 3.654 | ± | 0.100 |
| Coumaroylquinic acid (4) | 0.011 | ± | 0.001 |  | 0.011 | ± | 0.002 |  | 0.012 | ± | 0.001 |  | 0.008 | ± | 0.001 |  | 0.012 | ± | 0.001 |  | 0.010 | ± | 0.000 |  | 0.010 | ± | 0.001 |  | 0.011 | ± | 0.001 |
| *p*-Coumaric acid | 0.609 | ± | 0.274 |  | 0.594 | ± | 0.061 |  | 0.486 | ± | 0.105 |  | 0.288 | ± | 0.124 |  | 0.484 | ± | 0.134 |  | ND | | |  | 0.354 | ± | 0.207 |  | ND | | |
| Feruloylquinic acid (1) | 0.338 | ± | 0.021 |  | 0.349 | ± | 0.031 |  | 0.377 | ± | 0.052 |  | 0.360 | ± | 0.024 |  | 0.385 | ± | 0.081 |  | 0.332 | ± | 0.021 |  | 0.302 | ± | 0.039 |  | 0.324 | ± | 0.000 |
| Coumaroylquinic acid lactone | 0.155 | ± | 0.014 |  | 0.160 | ± | 0.024 |  | 0.168 | ± | 0.008 |  | 0.139 | ± | 0.024 |  | 0.150 | ± | 0.013 |  | 0.145 | ± | 0.021 |  | 0.148 | ± | 0.018 |  | 0.141 | ± | 0.001 |
| **Total** | 64.295 | ± | 5.736 |  | 65.234 | ± | 3.421 |  | 72.084 | ± | 2.807 |  | 59.530 | ± | 0.113 |  | 71.362 | ± | 5.679 |  | 66.658 | ± | 1.769 |  | 64.423 | ± | 2.044 |  | 63.035 | ± | 2.538 |
|  |  |  |  |  |  |  |  |  |  |  |  |  |  |  |  |  |  |  |  |  |  |  |  |  |  |  |  |  |  |  |  |
| **Hydroxycinnamic acid glycosides** |  |  |  |  |  |  |  |  |  |  |  |  |  |  |  |  |  |  |  |  |  |  |  |  |  |  |  |  |  |  |  |
| Coumaric acid-*O*-hexoside | 0.408 | ± | 0.033 |  | 0.700 | ± | 0.132 |  | 1.077 | ± | 0.099 |  | 0.378 | ± | 0.011 |  | 0.885 | ± | 0.076 |  | 1.082 | ± | 0.123 |  | 0.892 | ± | 0.254 |  | 1.162 | ± | 0.085 |
| Caffeic acid-*O*-hexoside | 0.211 | ± | 0.017 |  | 0.206 | ± | 0.005 |  | 0.237 | ± | 0.033 |  | 0.197 | ± | 0.002 |  | 0.232 | ± | 0.013 |  | 0.229 | ± | 0.008 |  | 0.211 | ± | 0.014 |  | 0.229 | ± | 0.020 |
| Caffeoylquinic acid-*O*-hexoside | 0.951 | ± | 0.050 |  | 0.796 | ± | 0.078 |  | 0.943 | ± | 0.061 |  | 0.777 | ± | 0.013 |  | 0.977 | ± | 0.072 |  | 0.826 | ± | 0.045 |  | 0.881 | ± | 0.045 |  | 0.877 | ± | 0.006 |
| **Total** | 1.570 | ± | 0.036 |  | 1.702 | ± | 0.185 |  | 2.258 | ± | 0.188 |  | 1.353 | ± | 0.026 |  | 2.093 | ± | 0.082 |  | 2.136 | ± | 0.098 |  | 1.984 | ± | 0.288 |  | 2.268 | ± | 0.064 |
|  |  |  |  |  |  |  |  |  |  |  |  |  |  |  |  |  |  |  |  |  |  |  |  |  |  |  |  |  |  |  |  |
| **Flavone** |  |  |  |  |  |  |  |  |  |  |  |  |  |  |  |  |  |  |  |  |  |  |  |  |  |  |  |  |  |  |  |
| Luteolin | 0.080 | ± | 0.029 |  | 0.204 | ± | 0.022 |  | 0.209 | ± | 0.025 |  | 0.087 | ± | 0.016 |  | 0.194 | ± | 0.019 |  | 0.193 | ± | 0.005 |  | 0.173 | ± | 0.008 |  | 0.175 | ± | 0.029 |
|  |  |  |  |  |  |  |  |  |  |  |  |  |  |  |  |  |  |  |  |  |  |  |  |  |  |  |  |  |  |  |  |
| **Hydroxybenzoic acids** |  |  |  |  |  |  |  |  |  |  |  |  |  |  |  |  |  |  |  |  |  |  |  |  |  |  |  |  |  |  |  |
| Dihydroxybenzoic acid *O*-hexoside | 2.085 | ± | 0.291 |  | 1.985 | ± | 0.185 |  | 2.393 | ± | 0.038 |  | 2.024 | ± | 0.177 |  | 2.453 | ± | 0.297 |  | 2.048 | ± | 0.281 |  | 1.943 | ± | 0.102 |  | 2.061 | ± | 0.163 |
| Protocatechuic acid | 0.457 | ± | 0.197 |  | 0.508 | ± | 0.023 |  | 0.382 | ± | 0.103 |  | 0.253 | ± | 0.114 |  | 0.380 | ± | 0.131 |  | ND | | |  | 0.308 | ± | 0.273 |  | ND | | |
| **Total** | 2.542 | ± | 0.327 |  | 2.493 | ± | 0.208 |  | 2.775 | ± | 0.107 |  | 2.277 | ± | 0.291 |  | 2.706 | ± | 0.535 |  | 2.048 | ± | 0.281 |  | 2.250 | ± | 0.367 |  | 2.061 | ± | 0.163 |
|  |  |  |  |  |  |  |  |  |  |  |  |  |  |  |  |  |  |  |  |  |  |  |  |  |  |  |  |  |  |  |  |
| **Flavonol** |  |  |  |  |  |  |  |  |  |  |  |  |  |  |  |  |  |  |  |  |  |  |  |  |  |  |  |  |  |  |  |
| Quercetin | 0.712 | ± | 0.058 |  | 1.335 | ± | 0.035 |  | 1.491 | ± | 0.121 |  | 0.657 | ± | 0.074 |  | 1.482 | ± | 0.105 |  | 1.339 | ± | 0.015 |  | 1.334 | ± | 0.066 |  | 1.375 | ± | 0.089 |
|  |  |  |  |  |  |  |  |  |  |  |  |  |  |  |  |  |  |  |  |  |  |  |  |  |  |  |  |  |  |  |  |
| **Flavonol glycosides** |  |  |  |  |  |  |  |  |  |  |  |  |  |  |  |  |  |  |  |  |  |  |  |  |  |  |  |  |  |  |  |
| Quercetin-3-*O*-rutinoside | 1.168 | ± | 0.029 |  | 1.269 | ± | 0.106 |  | 1.338 | ± | 0.065 |  | 0.938 | ± | 0.085 |  | 1.386 | ± | 0.127 |  | 1.206 | ± | 0.067 |  | 1.197 | ± | 0.095 |  | 1.241 | ± | 0.112 |
| Quercetin-3-*O*-glucoside | 0.099 | ± | 0.012 |  | 0.118 | ± | 0.007 |  | 0.127 | ± | 0.009 |  | 0.086 | ± | 0.005 |  | 0.131 | ± | 0.015 |  | 0.116 | ± | 0.006 |  | 0.112 | ± | 0.007 |  | 0.107 | ± | 0.001 |
| Kaempferol-*O*-rutinoside | 0.124 | ± | 0.007 |  | 0.133 | ± | 0.003 |  | 0.139 | ± | 0.006 |  | 0.105 | ± | 0.005 |  | 0.145 | ± | 0.019 |  | 0.134 | ± | 0.003 |  | 0.117 | ± | 0.010 |  | 0.128 | ± | 0.006 |
| **Total** | 1.391 | ± | 0.039 |  | 1.521 | ± | 0.105 |  | 1.604 | ± | 0.052 |  | 1.129 | ± | 0.095 |  | 1.662 | ± | 0.153 |  | 1.456 | ± | 0.071 |  | 1.427 | ± | 0.094 |  | 1.477 | ± | 0.106 |
|  |  |  |  |  |  |  |  |  |  |  |  |  |  |  |  |  |  |  |  |  |  |  |  |  |  |  |  |  |  |  |  |
| **Catechins** |  |  |  |  |  |  |  |  |  |  |  |  |  |  |  |  |  |  |  |  |  |  |  |  |  |  |  |  |  |  |  |
| (+)-Catechin | 0.227 | ± | 0.009 |  | 0.338 | ± | 0.033 |  | 0.568 | ± | 0.025 |  | 0.289 | ± | 0.045 |  | 0.453 | ± | 0.083 |  | 0.516 | ± | 0.056 |  | 0.483 | ± | 0.103 |  | 0.629 | ± | 0.024 |
| (-)-Epicatechin | 0.631 | ± | 0.046 |  | 0.956 | ± | 0.048 |  | 1.684 | ± | 0.294 |  | 0.801 | ± | 0.077 |  | 1.327 | ± | 0.089 |  | 1.576 | ± | 0.075 |  | 1.566 | ± | 0.204 |  | 1.992 | ± | 0.097 |
| **Total** | 0.858 | ± | 0.051 |  | 1.294 | ± | 0.060 |  | 2.252 | ± | 0.319 |  | 1.089 | ± | 0.122 |  | 1.780 | ± | 0.172 |  | 2.092 | ± | 0.100 |  | 2.049 | ± | 0.307 |  | 2.621 | ± | 0.118 |
|  |  |  |  |  |  |  |  |  |  |  |  |  |  |  |  |  |  |  |  |  |  |  |  |  |  |  |  |  |  |  |  |
| **Phenylpropionic acid** |  |  |  |  |  |  |  |  |  |  |  |  |  |  |  |  |  |  |  |  |  |  |  |  |  |  |  |  |  |  |  |
| Dihydrocaffeic acid | ND | | |  | ND | | |  | ND | | |  | ND | | |  | 0.307 | ± | 0.222 |  | 0.284 | ± | 0.043 |  | 0.131 | ± | 0.062 |  | 0.568 | ± | 0.070 |
|  |  |  |  |  |  |  |  |  |  |  |  |  |  |  |  |  |  |  |  |  |  |  |  |  |  |  |  |  |  |  |  |
| **Total phenolic compounds** | 71.449 | ± | 6.089 |  | 75.252 | ± | 3.974 |  | 83.331 | ± | 3.324 |  | 66.122 | ± | 0.161 |  | 84.913 | ± | 6.671 |  | 79.575 | ± | 1.593 |  | 76.230 | ± | 2.618 |  | 76.641 | ± | 2.709 |
|  |  |  |  |  |  |  |  |  |  |  |  |  |  |  |  |  |  |  |  |  |  |  |  |  |  |  |  |  |  |  |  |
|  |  |  |  |  |  |  |  |  |  |  |  |  |  |  |  |  |  |  |  |  |  |  |  |  |  |  |  |  |  |  |  |
|  | | | | | | | | | | | | | | | | | | | | | | | | | | | | | | | |
|  | | | | | | | | | | | | | | | | | | | | | | | | | | | | | | | |
| ***14 Days*** | | | | | | | | | | | | | | | | | | | | | | | | | | | | | | | |
|  | **37°C** | | |  | **2360** | | |  | **4186** | | |  | **30°C** | | |  | **1LE1** | | |  | **285** | | |  | **C1** | | |  | **POM1** | | |
|  |  |  |  |  |  |  |  |  |  |  |  |  |  |  |  |  |  |  |  |  |  |  |  |  |  |  |  |  |  |  |  |
| **Phenyllactic acids** |  |  |  |  |  |  |  |  |  |  |  |  |  |  |  |  |  |  |  |  |  |  |  |  |  |  |  |  |  |  |  |
| *p*-Hydroxyphenyllactic acid | ND | | |  | 1.272 | ± | 0.159 |  | 0.259 | ± | 0.030 |  | ND | | |  | 1.225 | ± | 0.025 |  | 1.473 | ± | 0.158 |  | 1.271 | ± | 0.115 |  | 1.295 | ± | 0.131 |
| Phenyllactic acid | ND | | |  | 0.559 | ± | 0.034 |  | 0.513 | ± | 0.006 |  | ND | | |  | 1.986 | ± | 0.087 |  | 2.150 | ± | 0.153 |  | 1.494 | ± | 0.141 |  | 2.208 | ± | 0.095 |
| **Total** | ND | | |  | 1.831 | ± | 0.190 |  | 0.772 | ± | 0.030 |  | ND | | |  | 3.211 | ± | 0.104 |  | 3.623 | ± | 0.264 |  | 2.765 | ± | 0.245 |  | 3.503 | ± | 0.222 |
|  |  |  |  |  |  |  |  |  |  |  |  |  |  |  |  |  |  |  |  |  |  |  |  |  |  |  |  |  |  |  |  |
| **Hydroxycinnamic acids** |  |  |  |  |  |  |  |  |  |  |  |  |  |  |  |  |  |  |  |  |  |  |  |  |  |  |  |  |  |  |  |
| 3-*O*-caffeoylquinic acid | 19.271 | ± | 0.407 |  | 17.498 | ± | 0.344 |  | 20.036 | ± | 1.511 |  | 18.976 | ± | 0.967 |  | 18.775 | ± | 2.710 |  | 17.786 | ± | 0.946 |  | 18.496 | ± | 1.410 |  | 18.544 | ± | 1.360 |
| 5-*O*-caffeoylquinic acid | 2.719 | ± | 0.180 |  | 2.979 | ± | 0.077 |  | 3.298 | ± | 0.100 |  | 2.700 | ± | 0.341 |  | 2.941 | ± | 0.220 |  | 3.309 | ± | 0.243 |  | 3.027 | ± | 0.343 |  | 2.930 | ± | 0.233 |
| 4-*O*-caffeoylquinic acid | 8.559 | ± | 0.784 |  | 8.395 | ± | 1.264 |  | 9.105 | ± | 0.907 |  | 7.859 | ± | 0.484 |  | 8.969 | ± | 0.828 |  | 8.933 | ± | 1.002 |  | 8.700 | ± | 0.489 |  | 8.984 | ± | 0.382 |
| Caffeoylquinic acid | 0.037 | ± | 0.006 |  | 0.042 | ± | 0.006 |  | 0.040 | ± | 0.005 |  | 0.036 | ± | 0.006 |  | 0.039 | ± | 0.008 |  | 0.041 | ± | 0.005 |  | 0.038 | ± | 0.001 |  | 0.039 | ± | 0.004 |
| Caffeic acid | 0.685 | ± | 0.062 |  | 0.789 | ± | 0.050 |  | 0.668 | ± | 0.038 |  | 0.702 | ± | 0.037 |  | 0.345 | ± | 0.241 |  | ND | | |  | 0.259 | ± | 0.024 |  | 0.092 | ± | 0.020 |
| Dicaffeoylquinic acid (1) | 0.010 | ± | 0.004 |  | 0.009 | ± | 0.001 |  | 0.010 | ± | 0.001 |  | 0.008 | ± | 0.001 |  | 0.007 | ± | 0.001 |  | 0.011 | ± | 0.001 |  | 0.008 | ± | 0.003 |  | 0.009 | ± | 0.003 |
| Dicaffeoylquinic acid (2) | 0.013 | ± | 0.005 |  | 0.022 | ± | 0.007 |  | 0.017 | ± | 0.006 |  | 0.011 | ± | 0.005 |  | 0.028 | ± | 0.005 |  | 0.025 | ± | 0.002 |  | 0.031 | ± | 0.005 |  | 0.033 | ± | 0.004 |
| Dicaffeoylquinic acid (3) | 0.029 | ± | 0.004 |  | 0.036 | ± | 0.001 |  | 0.032 | ± | 0.003 |  | 0.030 | ± | 0.004 |  | 0.038 | ± | 0.005 |  | 0.043 | ± | 0.007 |  | 0.041 | ± | 0.002 |  | 0.031 | ± | 0.005 |
| Coumaroylquinic acid (1) | 21.825 | ± | 0.576 |  | 23.674 | ± | 0.672 |  | 23.440 | ± | 1.247 |  | 22.012 | ± | 2.190 |  | 22.976 | ± | 0.759 |  | 22.844 | ± | 1.427 |  | 24.561 | ± | 1.478 |  | 24.533 | ± | 0.303 |
| Coumaroylquinic acid (2) | 6.936 | ± | 0.426 |  | 6.450 | ± | 0.212 |  | 6.768 | ± | 0.315 |  | 6.370 | ± | 1.109 |  | 6.816 | ± | 0.141 |  | 7.116 | ± | 0.461 |  | 6.765 | ± | 0.102 |  | 7.412 | ± | 0.704 |
| Coumaroylquinic acid (3) | 3.987 | ± | 0.214 |  | 4.000 | ± | 0.273 |  | 4.212 | ± | 0.152 |  | 3.803 | ± | 0.464 |  | 4.056 | ± | 0.139 |  | 4.073 | ± | 0.268 |  | 3.955 | ± | 0.266 |  | 3.735 | ± | 0.150 |
| Coumaroylquinic acid (4) | 0.012 | ± | 0.001 |  | 0.011 | ± | 0.001 |  | 0.011 | ± | 0.001 |  | 0.011 | ± | 0.001 |  | 0.010 | ± | 0.001 |  | 0.012 | ± | 0.000 |  | 0.011 | ± | 0.001 |  | 0.011 | ± | 0.000 |
| *p*-Coumaric acid | 0.670 | ± | 0.294 |  | 0.472 | ± | 0.050 |  | 0.485 | ± | 0.126 |  | 0.412 | ± | 0.103 |  | 0.337 | ± | 0.019 |  | ND | | |  | ND | | |  | ND | | |
| Feruloylquinic acid (1) | 0.340 | ± | 0.040 |  | 0.302 | ± | 0.038 |  | 0.339 | ± | 0.042 |  | 0.364 | ± | 0.047 |  | 0.376 | ± | 0.047 |  | 0.325 | ± | 0.037 |  | 0.323 | ± | 0.046 |  | 0.345 | ± | 0.016 |
| Coumaroylquinic acid lactone | 0.144 | ± | 0.004 |  | 0.177 | ± | 0.031 |  | 0.166 | ± | 0.016 |  | 0.143 | ± | 0.005 |  | 0.131 | ± | 0.032 |  | 0.149 | ± | 0.023 |  | 0.140 | ± | 0.002 |  | 0.126 | ± | 0.009 |
| **Total** | 65.236 | ± | 0.952 |  | 64.858 | ± | 1.759 |  | 68.627 | ± | 2.251 |  | 63.437 | ± | 5.218 |  | 65.732 | ± | 2.909 |  | 64.668 | ± | 0.863 |  | 66.355 | ± | 2.513 |  | 66.826 | ± | 2.637 |
|  |  |  |  |  |  |  |  |  |  |  |  |  |  |  |  |  |  |  |  |  |  |  |  |  |  |  |  |  |  |  |  |
| **Hydroxycinnamic acid glycosides** |  |  |  |  |  |  |  |  |  |  |  |  |  |  |  |  |  |  |  |  |  |  |  |  |  |  |  |  |  |  |  |
| Coumaric acid-*O*-hexoside | 0.541 | ± | 0.026 |  | 0.516 | ± | 0.133 |  | 0.621 | ± | 0.136 |  | 0.446 | ± | 0.170 |  | 0.480 | ± | 0.014 |  | 0.790 | ± | 0.020 |  | 0.732 | ± | 0.174 |  | 1.145 | ± | 0.142 |
| Caffeic acid-*O*-hexoside | 0.213 | ± | 0.005 |  | 0.198 | ± | 0.003 |  | 0.215 | ± | 0.003 |  | 0.211 | ± | 0.011 |  | 0.215 | ± | 0.009 |  | 0.213 | ± | 0.010 |  | 0.217 | ± | 0.007 |  | 0.204 | ± | 0.018 |
| Caffeoylquinic acid *O*-hexoside | 0.901 | ± | 0.086 |  | 0.785 | ± | 0.086 |  | 0.788 | ± | 0.056 |  | 0.781 | ± | 0.053 |  | 0.882 | ± | 0.044 |  | 0.882 | ± | 0.065 |  | 0.898 | ± | 0.056 |  | 0.868 | ± | 0.043 |
| **Total** | 1.655 | ± | 0.073 |  | 1.500 | ± | 0.056 |  | 1.623 | ± | 0.123 |  | 1.438 | ± | 0.224 |  | 1.576 | ± | 0.043 |  | 1.885 | ± | 0.095 |  | 1.847 | ± | 0.162 |  | 2.217 | ± | 0.188 |
|  |  |  |  |  |  |  |  |  |  |  |  |  |  |  |  |  |  |  |  |  |  |  |  |  |  |  |  |  |  |  |  |
| **Flavone** |  |  |  |  |  |  |  |  |  |  |  |  |  |  |  |  |  |  |  |  |  |  |  |  |  |  |  |  |  |  |  |
| Luteolin | 0.154 | ± | 0.012 |  | 0.175 | ± | 0.030 |  | 0.185 | ± | 0.021 |  | 0.129 | ± | 0.008 |  | 0.176 | ± | 0.011 |  | 0.157 | ± | 0.024 |  | 0.165 | ± | 0.014 |  | 0.166 | ± | 0.011 |
|  |  |  |  |  |  |  |  |  |  |  |  |  |  |  |  |  |  |  |  |  |  |  |  |  |  |  |  |  |  |  |  |
| **Hydroxybenzoic acids** |  |  |  |  |  |  |  |  |  |  |  |  |  |  |  |  |  |  |  |  |  |  |  |  |  |  |  |  |  |  |  |
| Dihydroxybenzoic acid *O*-hexoside | 2.116 | ± | 0.067 |  | 2.202 | ± | 0.179 |  | 2.336 | ± | 0.311 |  | 2.040 | ± | 0.426 |  | 2.223 | ± | 0.123 |  | 2.228 | ± | 0.046 |  | 1.894 | ± | 0.179 |  | 2.157 | ± | 0.104 |
| Protocatechuic acid | 0.560 | ± | 0.290 |  | 0.484 | ± | 0.170 |  | 0.497 | ± | 0.217 |  | 0.357 | ± | 0.148 |  | 0.285 | ± | 0.052 |  | ND | | |  | 0.459 | ± | 0.247 |  | ND | | |
| **total** | 2.676 | ± | 0.329 |  | 2.686 | ± | 0.315 |  | 2.833 | ± | 0.381 |  | 2.397 | ± | 0.568 |  | 2.413 | ± | 0.142 |  | 2.228 | ± | 0.046 |  | 2.353 | ± | 0.296 |  | 2.157 | ± | 0.104 |
|  |  |  |  |  |  |  |  |  |  |  |  |  |  |  |  |  |  |  |  |  |  |  |  |  |  |  |  |  |  |  |  |
| **Flavonol** |  |  |  |  |  |  |  |  |  |  |  |  |  |  |  |  |  |  |  |  |  |  |  |  |  |  |  |  |  |  |  |
| Quercetin | 1.091 | ± | 0.036 |  | 1.353 | ± | 0.038 |  | 1.333 | ± | 0.072 |  | 0.957 | ± | 0.075 |  | 1.339 | ± | 0.020 |  | 1.353 | ± | 0.056 |  | 1.332 | ± | 0.036 |  | 1.348 | ± | 0.093 |
|  |  |  |  |  |  |  |  |  |  |  |  |  |  |  |  |  |  |  |  |  |  |  |  |  |  |  |  |  |  |  |  |
| **Flavonols glycosides** |  |  |  |  |  |  |  |  |  |  |  |  |  |  |  |  |  |  |  |  |  |  |  |  |  |  |  |  |  |  |  |
| Quercetin-3-*O*-rutinoside | 1.270 | ± | 0.064 |  | 1.315 | ± | 0.127 |  | 1.285 | ± | 0.117 |  | 1.284 | ± | 0.168 |  | 1.146 | ± | 0.063 |  | 1.184 | ± | 0.267 |  | 1.250 | ± | 0.079 |  | 1.335 | ± | 0.026 |
| Quercetin-3-*O*-glucoside | 0.118 | ± | 0.005 |  | 0.126 | ± | 0.006 |  | 0.127 | ± | 0.015 |  | 0.109 | ± | 0.014 |  | 0.117 | ± | 0.009 |  | 0.125 | ± | 0.011 |  | 0.123 | ± | 0.007 |  | 0.119 | ± | 0.012 |
| Kaempferol-*O*-rutinoside | 0.136 | ± | 0.003 |  | 0.123 | ± | 0.005 |  | 0.139 | ± | 0.009 |  | 0.114 | ± | 0.009 |  | 0.125 | ± | 0.007 |  | 0.132 | ± | 0.008 |  | 0.127 | ± | 0.009 |  | 0.126 | ± | 0.007 |
| **Total** | 1.524 | ± | 0.071 |  | 1.564 | ± | 0.128 |  | 1.551 | ± | 0.141 |  | 1.508 | ± | 0.185 |  | 1.388 | ± | 0.068 |  | 1.441 | ± | 0.278 |  | 1.500 | ± | 0.078 |  | 1.580 | ± | 0.019 |
|  |  |  |  |  |  |  |  |  |  |  |  |  |  |  |  |  |  |  |  |  |  |  |  |  |  |  |  |  |  |  |  |
| **Catechins** |  |  |  |  |  |  |  |  |  |  |  |  |  |  |  |  |  |  |  |  |  |  |  |  |  |  |  |  |  |  |  |
| (+)-Catechin | 0.195 | ± | 0.020 |  | 0.192 | ± | 0.028 |  | 0.291 | ± | 0.011 |  | 0.212 | ± | 0.047 |  | 0.291 | ± | 0.029 |  | 0.390 | ± | 0.035 |  | 0.332 | ± | 0.057 |  | 0.461 | ± | 0.014 |
| (-)-Epicatechin | 0.424 | ± | 0.107 |  | 0.532 | ± | 0.171 |  | 0.744 | ± | 0.043 |  | 0.534 | ± | 0.080 |  | 0.849 | ± | 0.120 |  | 0.984 | ± | 0.125 |  | 1.108 | ± | 0.135 |  | 1.360 | ± | 0.039 |
| **Total** | 0.619 | ± | 0.117 |  | 0.724 | ± | 0.188 |  | 1.035 | ± | 0.052 |  | 0.746 | ± | 0.127 |  | 1.140 | ± | 0.111 |  | 1.374 | ± | 0.158 |  | 1.440 | ± | 0.108 |  | 1.821 | ± | 0.052 |
|  |  |  |  |  |  |  |  |  |  |  |  |  |  |  |  |  |  |  |  |  |  |  |  |  |  |  |  |  |  |  |  |
| **Phenylpropionic acids** |  |  |  |  |  |  |  |  |  |  |  |  |  |  |  |  |  |  |  |  |  |  |  |  |  |  |  |  |  |  |  |
| Dihydrocaffeic acid | ND | | |  | ND | | |  | ND | | |  | ND | | |  | 0.317 | ± | 0.314 |  | 0.278 | ± | 0.022 |  | 0.124 | ± | 0.040 |  | 0.609 | ± | 0.041 |
|  |  |  |  |  |  |  |  |  |  |  |  |  |  |  |  |  |  |  |  |  |  |  |  |  |  |  |  |  |  |  |  |
| **Total phenolic compounds** | 72.955 | ± | 0.904 |  | 74.691 | ± | 2.298 |  | 77.960 | ± | 2.585 |  | 70.611 | ± | 6.185 |  | 77.293 | ± | 3.104 |  | 77.007 | ± | 0.879 |  | 77.881 | ± | 2.910 |  | 80.226 | ± | 2.548 |

ND. Not detected
